# Health-related quality of life in seven European countries throughout the course of the COVID-19 pandemic: evidence from the European COvid Survey (ECOS)

**DOI:** 10.1007/s11136-022-03334-5

**Published:** 2023-02-05

**Authors:** Hans-Helmut König, Sebastian Neumann-Böhme, Iryna Sabat, Jonas Schreyögg, Aleksandra Torbica, Job van Exel, Pedro Pita Barros, Tom Stargardt, André Hajek

**Affiliations:** 1 Department of Health Economics and Health Services Research, University Medical Center Hamburg-Eppendorf, Hamburg Center for Health Economics, Martinistr. 52, 20246 Hamburg, Germany; 2 Erasmus School of Health Policy & Management, Erasmus University Rotterdam, Rotterdam, The Netherlands; 3 Hamburg Center for Health Economics, University of Hamburg, Hamburg, Germany; 4 Nova School of Business and Economics, Carcavelos, Portugal; 5 Department of Social and Political Sciences, SDA Bocconi School of Management, Bocconi University, Milan, Italy

**Keywords:** Health-related quality of life, EQ-5D, COVID-19, Corona, SARS-CoV-2

## Abstract

**Purpose:**

To investigate health-related quality of life (HRQoL) over the course of the COVID-19 pandemic in seven European countries and its association with selected sociodemographic as well as COVID-19-related variables.

**Methods:**

We used longitudinal data from nine quarterly waves collected between April 2020 and January 2022 (sample size per wave ranging from *N* = 7025 to 7300) of the European COvid Survey (ECOS), a representative survey of adults in Germany, United Kingdom, Denmark, Netherlands, France, Portugal and Italy. HRQoL was measured using the EQ-5D-5L. The association of self-reported COVID-19 infection, perceived health risk from COVID-19, selected sociodemographic variables and the COVID-19 stringency index with HRQoL was analyzed by logistic and linear fixed effects regressions.

**Results:**

On average across all nine waves, the proportion of respondents reporting any problems in at least one of the EQ-5D dimensions ranged between 63.8% (Netherlands) and 71.0% (Denmark). Anxiety/depression was the most frequently affected EQ-5D dimension in four countries (Portugal: 52.0%; United Kingdom: 50.2%; Italy: 49.2%; France: 49.0%), whereas pain/discomfort ranked first in three countries (Denmark: 58.3%; Germany: 55.8%; Netherlands: 49.0%). On average across all nine waves, the EQ-VAS score ranged from 70.1 in the United Kingdom to 78.4 in Portugal. Moreover, the EQ-5D-5L index ranged from .82 in Denmark to .94 in France. The occurrence of COVID-19 infection, changes in the perceived risk to one’s own health from COVID-19, the occurrence of income difficulties and an increase in the COVID-19 stringency index were associated with increased likelihood of problems in EQ-5D dimensions, reduced EQ-VAS score and reduced EQ-5D-5L index.

**Conclusions:**

Across seven European countries, we found large proportions of respondents reporting problems in HRQoL dimensions throughout the pandemic, especially for anxiety/depression. Various sociodemographic and COVID-19-related variables were associated with HRQoL in longitudinal analysis.

**Supplementary Information:**

The online version contains supplementary material available at 10.1007/s11136-022-03334-5.

## Introduction

The COVID-19 pandemic has affected people’s physical, mental and social health in numerous ways. First of all, people who contract COVID-19 may suffer from physical impairment caused by disease symptoms [1], social impairment caused by isolation or quarantine, and mental impairment due to cognitive or psychiatric symptoms, worries about recovery, social exclusion or stigma [2]. Secondly, people may suffer mentally from worries about risk of infection for themselves, family members or community members [3]. Thirdly, people may suffer from the social and economic consequences of the measures taken by governments against the spread of COVID-19 by restricting social contacts, such as closure of schools or businesses. While the latter may clearly affect mental and social aspects of health in the general population [4], effects on physical health are also possible, e.g. due to lack of physical activity [5] or underuse of preventive or curative health services [6].

The subjective assessment of physical, mental and social dimensions of health is commonly referred to by the concept of health-related quality of life (HRQoL) [7]. The EQ-5D is a family of generic instruments for measuring HRQoL (www.euroqol.org), available in numerous languages. Compared to other generic HRQoL instruments (e.g. SF-36), the EQ-5D instruments are very short and simple, and therefore easy to apply in large surveys. During the last 30 years, EQ-5D instruments have been used to measure the HRQoL of general population and patient samples in numerous countries.

Although the impact of the COVID-19 pandemic on people’s health is likely to be substantial, so far only few studies have assessed the impact on HRQoL and its determinants in general population samples, and only very few studies were conducted longitudinally or simultaneously in several countries. A cross-sectional general population survey (*N* = 4855) conducted in the USA in April 2020 reported significantly reduced HRQoL measured by the visual analog scale of the EQ-5D (EQ-VAS) as compared to established US normative data [8]. A repeated cross-sectional general population survey conducted in Alberta, Canada, in May/June 2020 (*N* = 8790) and October 2020 (*N* = 9263) found lower HRQoL measured by the EQ-5D-5L instrument compared to pre-pandemic surveys in this population, with the dimensions anxiety/depression and usual activities affected the most [9]. Another cross-sectional study conducted in general population samples in eight countries (*N* = 21,352) from April to June 2020 found that the stringency of government response in terms of restricting social contacts was associated with a small increase in EQ-5D-5L scores [10]. Finally, a longitudinal Japanese study conducted from January 2020 to February 2021 (*N* = 826) reported a decline in both physical and mental dimensions of HRQoL measured by the SF-36 instrument from immediately before the COVID-19 outbreak to one year later, in particular in women and respondents with lower economic status or lower general health [11].

In summary, the limited number of existing studies point at impaired HRQoL in the general population during the pandemic, with the mental dimension of HRQoL being particularly affected. While worries about risk of infection seem to be associated with reduced HRQoL, one cross-country comparison points at the stringency of government response possibly being positively associated with HRQoL. Yet, to our knowledge there are only few cross-country comparisons and longitudinal studies investigating the development of HRQoL and its determinants throughout the pandemic, considering fluctuations over time in e.g., the perceived risk of infection or the stringency of government response. Therefore, the purpose of this study was to describe HRQoL measured by the EQ-5D-5L in seven European countries over the course of the pandemic and to analyze longitudinally the impact of selected sociodemographic as well as COVID-19-related variables, namely experience with infection, perceived risk of COVID-19 for oneself and others, and stringency of government measures against the spread of COVID-19.

## Methods

### Sample

Longitudinal data were taken from nine waves of the European COvid Survey (ECOS) (wave 1 in April 2020: *N* = 7160; wave 2 in June 2020: *N* = 7122; wave 3 in September 2020: *N* = 7025; wave 4 in November 2020: *N* = 7115; wave 5 in January/February 2021: *N* = 7068; wave 6 in April 2021: *N* = 7204; wave 7 in June/July 2021: *N* = 7073; wave 8 in September 2021: *N* = 7232; and wave 9 in December 2021/January 2022: *N* = 7300), including samples from Germany, the United Kingdom, Denmark, the Netherlands, France, Portugal, and Italy.

Dynata, a market research company, collected data from online panels of about 1,000 adult individuals in each country and in each wave. To reach the general population, several recruiting techniques were used (i.e., open recruitment, loyalty programs, mobile apps or affiliate networks). Quota sampling was used to ensure representativeness in terms of gender, six age categories (18–24, 25–34, 35–44, 45–54, 55–64, and 65 + years), region, and education (all non-interlocked) in each country separately using national census data for quota. Refreshment samples were drawn. In each wave, former respondents were invited first. New respondents (i.e., refreshment samples) were invited only after several reminders to ensure representativeness. Reminders were not sent manually but by an electronic tool set up by Dynata called “Picker” which allows to control the sampling speed and the quota performance by increasing or slowing down the progress for the recontacts and the refreshment samples. About 47% of all respondents were followed over two or more waves. Sabat et al. provided additional details [12]. No further exclusion criteria were applied.

Each participant provided written informed consent to Dynata. The confidentiality and anonymity of the participants were ensured. This study received ethical approval from the University of Hamburg in Germany (under the umbrella project "Countering COVID-19: A European survey on the acceptability and commitment to preventive measures").

#### EQ-5D-5L

The EQ-5D-5L questionnaire consists of five items referring to current problems in the HRQoL dimensions ‘mobility’, ‘self-care’, ‘usual activities’, ‘pain/discomfort’, and ‘anxiety/depression’ [13]. Each item has five response levels: no problems, slight problems, moderate problems, severe problems and extreme problems. This part of the questionnaire is called EQ-5D descriptive system and provides a profile of HRQoL. Due to skewness of data and for simplification, the five items were dichotomized (0 = no problems in the respective dimension; 1 = problems in the respective dimension (including slight problems, moderate problems, severe problems and extreme problems) for description and regression analysis of problem frequency. Additionally, a binary variable was computed for regression analysis (1 = problems in any dimension; 0 = otherwise).

Furthermore, the questionnaire includes a visual analog scale (known as EQ-VAS). The EQ-VAS records self-rated health based on the respondent’s preferences, with a scale ranging from 0 (indicating the worst imaginable health) to 100 (indicating the best imaginable health).

Moreover, the HRQoL profile provided by the EQ-5D descriptive system can be converted into an index value (EQ-5D index) based on country-specific value sets representing societal preferences. Standard EQ-5D value sets have been obtained from representative samples of the general public in numerous countries using a standardized valuation technique with the best health state (no problems in any EQ-5D dimension) and death being assigned values of 1 and 0, respectively. Accordingly, we calculated the country-specific EQ-5D-5L index based on standard value sets available for the included countries [14–20].

### Sociodemographic and COVID-19-related variables

Besides the time-constant variables sex (men or women) and country (Germany; United Kingdom; Denmark; Netherlands; France; Portugal; Italy) we used the following time-varying sociodemographic and COVID-19-related variables for the analyses: age, level of education (three categories: low education; medium education; high education; based on the country specific education system; for additional details please see Varghese et al. [21]), self-assessed difficulties with income (“Thinking of your household's total monthly income, would you say that your household is able to make ends meet… “: with great difficulty; with some difficulties; fairly easily; easily), professional group (five sectors: health-related sector; education; food retail; research; other), experience of infection with the coronavirus (four categories: no; yes, confirmed; yes, but not yet confirmed; don’t know), one’s own perceived risk of getting infected with the coronavirus (single item from 1 = no risk at all to 5 = very high risk), perceived risk to one’s own health from COVID-19 (single item from 1 = no risk at all to 5 = very high risk), perceived risk to the health of family members from COVID-19 (single item from 1 = no risk at all to 5 = very high risk), and perceived risk to the health of people in own community from COVID-19 (single item from 1 = no risk at all to 5 = very high risk). Marital status (married/registered partnership; living together (relationship); living alone (single); living alone (in a relationship); widowed; other) was only included from wave 3 onwards. In addition, we added country-specific and ECOS wave-specific data from the COVID-19 stringency index of the Oxford Covid-19 Government Response Tracker (OxCGRT) [22]. This index is a proxy for the strictness of government response aimed at reducing social contacts by restricting people’s behavior. It is a composite measure based on nine indicators including school closures, workplace closures, cancellation of public events, restrictions on public gatherings, closures of public transport, stay-at-home requirements, public information campaigns, restrictions on internal movements, and international travel controls. The index ranges from 0 to 100, with higher scores indicating stricter measures in place. If policies vary at the subnational level, the index is indicated as the response level of the strictest sub-region.

As descriptive context variable, we also report the number of daily new confirmed COVID-19 cases per million people (7-day rolling average) by country and by wave (https://ourworldindata.org/).

### Statistical analysis

In a first step, sample characteristics at baseline (i.e., wave 1) stratified by country are shown to get a first impression of the data used. Subsequently, frequency of problems in the five EQ-5D-5L dimensions (plus problems in any dimension) as well as EQ-VAS score and EQ-5D-5L index are presented stratified by country and wave. Lastly, sociodemographic and COVID-19-related determinants of problems in the five EQ-5D-5L dimensions (plus problems in any dimension), of the EQ-VAS score and the EQ-5D-5L index were estimated using conditional fixed effects (FE) logistic regressions or linear FE regressions, as appropriate.

Using FE regressions can assist in investigating within-information over time—which corresponds to examining changes within participants over time [23]. A key advantage of FE regressions is that they produce consistent estimates under quite weak assumptions (e.g., allowing for a correlation between time-constant factors and the regressors)—for example, compared to random-effects regressions [23]. Our approach was substantiated by Sargan-Hansen tests (e.g., with EQ-VAS as outcome measure, Sargan-Hansen statistic was 995.8, p < 0.001) [24].

The FE regressions solely used changes within participants (i.e., intraindividual changes) over time (wave 1 to wave 9). For example, intraindividual changes in EQ-VAS scores can be examined. This also means that only time-varying sociodemographic and COVID-19-related variables factors (e.g., perceived income difficulties) can be used as independent variables, whereas time-constant factors (sex and country or region) cannot be included in FE regressions as main effects. While it should be noted that the FE estimates exclusively refer to participants who reported changes in dependent and independent in the observation period, it should also be emphasized that this is not a shortcoming of the analytical approach. It rather simply mirrors the fact that not all individuals had such changes in the observation period. Thus, an average treatment effect on the treated is estimated [25]. It should be noted that individuals who participated at multiple (but not all) waves are also included in FE regressions (as long as they have intraindividual changes over time).

With regard to missing data: While most of the variables (e.g., problems in the EQ-5D dimensions or sociodemographic factors) did not have missing data at all, the highest proportion of missing data in the total sample in wave 1 was identified for educational level (1.5%). Roughly similar proportions of missing values were identified for the other waves. Therefore, listwise deletion was used in this study.

The significance level was set at *α* = 0.05. To conduct statistical analysis, Stata 16.1 was used. In sensitivity analysis, we also included marital status which was only assessed from wave 3 onwards.

## Results

### Sample characteristics

Sample characteristics at baseline (wave 1, April 2020) stratified by country are shown in Table 1. Mean age ranged from 43.8 years in Portugal to 48.6 years in Germany, and the proportion of females from 51.0% in the Netherlands to 52.5% in Portugal. Between 15.4% (Denmark) and 40.9% (Portugal) of respondents reported a low educational level, and between 5.8% (Italy) and 13.7% (Denmark) worked in a health-related sector. Between 39.8% (Denmark) and 61.5% (Italy) of respondents reported some or great perceived difficulties with income. Only between 1.1% (Portugal) and 8.1% (United Kingdom) reported an infection (confirmed or unconfirmed) with the novel coronavirus at baseline. Mean scores for perceived risk of getting infected with the coronavirus ranged from 2.6 (Portugal) to 3.1 (United Kingdom), for perceived risk to one’s own health from 2.8 (Portugal and Denmark) to 3.2 (United Kingdom and France), for perceived risk to the health of one’s own family members from 3.1 (Denmark) to 3.5 (France) and for perceived risk to the health of people in one’s own community from 3.0 (Demark) to 3.4 (United Kingdom and Italy). The countries’ COVID-19 stringency index ranged from 72.0 (Denmark) to 88.6 (Italy), and the number of daily new confirmed COVID-19 cases per million people (7-day rolling average) ranged from 31.1 in Denmark to 171.3 in France. Additionally, sample characteristics for all nine waves (among the total sample) are shown in Supplementary Table 1. Most notably, in the total sample the proportion of respondents reporting an infection (confirmed or unconfirmed) increased from 4.3% in wave 1 to 14.9% in wave 9, the mean stringency index oscillated between 81.3 in wave 1 and 50.4 in wave 8, and the mean number of daily new confirmed COVID-19 cases per million people from 10.9 in wave 2 to 1800.9 in wave 9.

**Table 1:** Sample characteristics at wave 1 stratified by country

	Germany	United Kingdom	Denmark	Netherlands	France	Portugal	Italy
	*N* = 1000	*N* = 1009	*N* = 1000	*N* = 1012	*N* = 1000	*N* = 1064	*N* = 1075
Age	48.6 (16.4)	46.5 (16.8)	47.9 (16.9)	47.5 (16.7)	47.0 (16.7)	43.8 (15.7)	48.3 (16.2)
Gender							
Male	482 (48.2%)	482 (47.8%)	489 (48.9%)	496 (49.0%)	477 (47.7%)	505 (47.5%)	519 (48.3%)
Female	518 (51.8%)	527 (52.2%)	511 (51.1%)	516 (51.0%)	523 (52.3%)	559 (52.5%)	556 (51.7%)
Educational level							
Low	163 (16.6%)	290 (29.2%)	152 (15.4%)	327 (32.7%)	295 (30.2%)	428 (40.9%)	381 (35.8%)
Middle	596 (60.6%)	397 (40.0%)	554 (56.2%)	427 (42.7%)	401 (41.0%)	295 (28.2%)	472 (44.3%)
High	225 (22.9%)	306 (30.8%)	279 (28.3%)	247 (24.7%)	282 (28.8%)	323 (30.9%)	212 (19.9%)
Professional group							
Health-related sector	68 (6.8%)	86 (8.5%)	137 (13.7%)	107 (10.6%)	78 (7.8%)	76 (7.1%)	62 (5.8%)
Education	73 (7.3%)	86 (8.5%)	94 (9.4%)	53 (5.2%)	50 (5.0%)	78 (7.3%)	66 (6.1%)
Food retail	49 (4.9%)	78 (7.7%)	51 (5.1%)	50 (4.9%)	57 (5.7%)	71 (6.7%)	39 (3.6%)
Research	36 (3.6%)	14 (1.4%)	30 (3.0%)	26 (2.6%)	29 (2.9%)	31 (2.9%)	19 (1.8%)
Other	774 (77.4%)	745 (73.8%)	688 (68.8%)	776 (76.7%)	786 (78.6%)	808 (75.9%)	889 (82.7%)
Perceived income difficulties							
With great difficulty	113 (11.3%)	98 (9.7%)	86 (8.6%)	98 (9.7%)	130 (13.0%)	103 (9.7%)	191 (17.8%)
With some difficulty	400 (40.0%)	357 (35.4%)	312 (31.2%)	372 (36.8%)	475 (47.5%)	336 (31.6%)	470 (43.7%)
Fairly easily	365 (36.5%)	392 (38.9%)	400 (40.0%)	370 (36.6%)	330 (33.0%)	561 (52.7%)	352 (32.7%)
Easily	122 (12.2%)	162 (16.1%)	202 (20.2%)	172 (17.0%)	65 (6.5%)	64 (6.0%)	62 (5.8%)
Infection with the novel coronavirus							
Yes, confirmed	22 (2.2%)	31 (3.1%)	12 (1.2%)	18 (1.8%)	20 (2.0%)	6 (0.6%)	13 (1.2%)
Yes, but not confirmed by tests	13 (1.3%)	50 (5.0%)	29 (2.9%)	35 (3.5%)	40 (4.0%)	5 (0.5%)	16 (1.5%)
No	801 (80.1%)	804 (79.7%)	739 (73.9%)	679 (67.1%)	687 (68.7%)	762 (71.6%)	879 (81.8%)
Don’t know	164 (16.4%)	124 (12.3%)	220 (22.0%)	280 (27.7%)	253 (25.3%)	291 (27.3%)	167 (15.5%)
Own risk of getting infected with the coronavirus (from 1 = no risk at all to 5 = very high risk)	2.9 (1.1)	3.1 (1.1)	2.8 (1.0)	2.9 (1.0)	2.9 (1.1)	2.6 (1.0)	2.9 (1.1)
Risk to one’s own health from COVID-19 (from 1 = no risk at all to 5 = very high risk)	3.0 (1.2)	3.2 (1.2)	2.8 (1.2)	2.9 (1.2)	3.2 (1.2)	2.8 (1.2)	3.0 (1.2)
Risk to the health of one’s own family members from COVID-19 (from 1 = no risk at all to 5 = very high risk)	3.2 (1.2)	3.4 (1.2)	3.1 (1.2)	3.2 (1.1)	3.5 (1.2)	3.2 (1.3)	3.3 (1.3)
Risk to the health of people in one’s own community from COVID-19 (from 1 = no risk at all to 5 = very high risk)	3.2 (1.0)	3.4 (1.1)	3.0 (1.1)	3.1 (1.0)	3.3 (1.2)	3.1 (1.2)	3.4 (1.1)
COVID-19 stringency index (from 0 to 100, with 100 = strictest))	76.8 (0.0)	79.6 (0.0)	72.0 (0.0)	78.7 (0.0)	88.0 (0.0)	84.4 (0.0)	88.6 (0.0)
Daily new confirmed COVID-19 cases per million people (7-day rolling average)	41.7 (0.0)	63.1 (0.0)	31.1 (0.0)	60.7 (0.0)	171.3 (0.0)	68.9 (0,0)	62.3 (0.0)

Mean (SD) or N (%) are shown, as appropriate

Supplementary Table 2 compares country samples of all waves with the respective census populations in terms of age and gender (also providing the references for the census populations used). With the exception of too small proportions of respondents in the highest age category (65 +) in Portugal (in all waves) and Denmark (in wave 6), country samples were quite similar to the census populations. The average retention rate of participants, i.e., the share of participants in a wave that had also participated in the wave before, was 56.2% across all countries and waves, ranging from 38.3% in Portugal in wave 7 to 71.1% in Germany in wave 5.

### Health-related quality of life over the course of the pandemic

Table 2 presents the proportion of respondents reporting problems in the EQ-5D dimensions (dichotomized: no problems vs. at least slight problems) as well as mean EQ-VAS score and mean EQ-5D-5L-index, stratified by country and wave (wave 1 to wave 9). Furthermore, average proportions and scores across all nine waves are presented by country in the last column.

**Table 2:** Health-related quality of life (in terms of problems in the EQ-5D dimensions, EQ-VAS and EQ-5D-5L index) stratified by country and wave (wave 1 to wave 9)

	Wave 1	Wave 2	Wave 3	Wave 4	Wave 5	Wave 6	Wave 7	Wave 8	Wave 9	Total
Germany	*N* = 1000	*N* = 1050	*N* = 1005	*N* = 1043	*N* = 1008	*N* = 1013	*N* = 1015	*N* = 1027	*N* = 1007	*N* = 9168
EQ-VAS score: mean (SD)	73.4 (22.0)	72.7 (23.7)	70.9 (23.5)	69.4 (25.5)	70.2 (24.7)	70.5 (23.8)	69.4 (24.9)	70.7 (24.2)	70.1 (23.4)	70.8 (24.0)
EQ-5D-5L index: mean (SD)	0.86 (0.19)	0.86 (0.20)	0.85 (0.22)	0.84 (0.22)	0.85 (0.21)	0.85 (0.21)	0.85 (0.22)	0.85 (0.21)	0.84 (0.23)	0.85 (0.21)
Mobility: any problems *N* (%)	339 (33.9%)	382 (36.4%)	367 (36.5%)	395 (37.9%)	381 (37.8%)	346 (34.2%)	380 (37.4%)	372 (36.2%)	365 (36.2%)	3327 (36.3%)
Self-care: any problems *N* (%)	116 (11.6%)	125 (11.9%)	162 (16.1%)	183 (17.5%)	187 (18.6%)	172 (17.0%)	182 (17.9%)	181 (17.6%)	182 (18.1%)	1490 (16.3%)
Usual activities: any problems *N* (%)	285 (28.5%)	289 (27.5%)	268 (26.7%)	307 (29.4%)	305 (30.3%)	284 (28.0%)	290 (28.6%)	290 (28.2%)	284 (28.2%)	2602 (28.4%)
Pain/discomfort: any problems *N* (%)	586 (58.6%)	605 (57.6%)	536 (53.3%)	596 (57.1%)	570 (56.5%)	523 (51.6%)	528 (52.0%)	586 (57.1%)	585 (58.1%)	5115 (55.8%)
Anxiety/depression: any problems *N* (%)	474 (47.4%)	420 (40.0%)	409 (40.7%)	467 (44.8%)	438 (43.5%)	429 (42.3%)	388 (38.2%)	401 (39.0%)	399 (39.6%)	3825 (41.7%)
Any dimension: any problems *N* (%)	746 (74.6%)	746 (71.0%)	681 (67.8%)	732 (70.2%)	709 (70.3%)	676 (66.7%)	673 (66.3%)	692 (67.4%)	694 (68.9%)	6349 (69.3%)

United Kingdom	*N* = 1009	*N* = 1041	*N* = 1005	*N* = 1006	*N* = 1016	*N* = 1024	*N* = 1019	*N* = 1038	*N* = 1023	*N* = 9181
EQ-VAS score: mean (SD)	71.0 (23.3)	73.1 (22.5)	72.4 (22.0)	70.4 (23.9)	67.9 (24.1)	68.7 (24.7)	69.5 (24.5)	68.5 (25.2)	69.4 (23.7)	70.1 (23.8)
EQ-5D-5L index: mean (SD)	0.83 (0.21)	0.84 (0.19)	0.85 (0.20)	0.83 (0.21)	0.83 (0.21)	0.83 (0.22)	0.82 (0.22)	0.81 (0.23)	0.83 (0.22)	0.83 (0.21)
Mobility: any problems *N* (%)	281 (27.8%)	264 (25.4%)	257 (25.6%)	288 (28.6%)	281 (27.7%)	297 (29.0%)	308 (30.2%)	319 (30.7%)	286 (28.0%)	2581 (28.1%)
Self-care: any problems *N* (%)	144 (14.3%)	150 (14.4%)	143 (14.2%)	158 (15.7%)	175 (17.2%)	183 (17.9%)	225 (22.1%)	232 (22.4%)	190 (18.6%)	1600 (17.4%)
Usual activities: any problems *N* (%)	297 (29.4%)	282 (27.1%)	251 (25.0%)	284 (28.2%)	290 (28.5%)	292 (28.5%)	316 (31.0%)	333 (32.1%)	279 (27.3%)	2624 (28.6%)
Pain/discomfort: any problems *N* (%)	493 (48.9%)	523 (50.2%)	442 (44.0%)	475 (47.2%)	500 (49.2%)	452 (44.1%)	478 (46.9%)	502 (48.4%)	475 (46.4%)	4340 (47.3%)
Anxiety/depression: any problems *N* (%)	564 (55.9%)	533 (51.2%)	494 (49.2%)	531 (52.8%)	541 (53.2%)	463 (45.2%)	492 (48.3%)	499 (48.1%)	491 (48.0%)	4608 (50.2%)
Any dimension: any problems *N* (%)	732 (72.5%)	747 (71.8%)	657 (65.4%)	682 (67.8%)	725 (71.4%)	650 (63.5%)	667 (65.5%)	680 (65.5%)	661 (64.6%)	6201 (67.5%)

Denmark	*N* = 1000	*N* = 1005	*N* = 1000	*N* = 1012	*N* = 1012	*N* = 1023	*N* = 1008	*N* = 1035	*N* = 1016	*N* = 9111
EQ-VAS score: mean (SD)	76.4 (21.2)	74.2 (22.1)	72.4 (23.4)	72.5 (23.8)	72.5 (21.9)	73.3 (22.7)	71.5 (23.4)	72.1 (23.8)	72.4 (23.1)	73.0 (22.9)
EQ-5D-5L index: mean (SD)	0.84 (0.23)	0.82 (0.24)	0.81 (0.26)	0.80 (0.26)	0.80 (0.26)	0.82 (0.26)	0.81 (0.26)	0.82 (0.25)	0.82 (0.23)	0.82 (0.25)
Mobility: any problems N (%)	307 (30.7%)	338 (33.6%)	314 (31.4%)	338 (33.4%)	355 (35.1%)	314 (30.7%)	333 (33.0%)	333 (32.2%)	331 (32.6%)	2963 (32.5%)
Self-care: any problems N (%)	113 (11.3%)	135 (13.4%)	146 (14.6%)	155 (15.3%)	155 (15.3%)	141 (13.8%)	167 (16.6%)	161 (15.6%)	157 (15.5%)	1330 (14.6%)
Usual activities: any problems *N* (%)	344 (34.4%)	369 (36.7%)	322 (32.2%)	353 (34.9%)	376 (37.2%)	334 (32.6%)	346 (34.3%)	350 (33.8%)	344 (33.9%)	3138 (34.4%)
Pain/discomfort: any problems *N* (%)	566 (56.6%)	631 (62.8%)	579 (57.9%)	597 (59.0%)	603 (59.6%)	586 (57.3%)	586 (58.1%)	581 (56.1%)	579 (57.0%)	5308 (58.3%)
Anxiety/depression: any problems *N* (%)	365 (36.5%)	382 (38.0%)	380 (38.0%)	433 (42.8%)	419 (41.4%)	379 (37.0%)	413 (41.0%)	402 (38.8%)	404 (39.8%)	3577 (39.3%)
Any dimension: any problems N (%)	694 (69.4%)	748 (74.4%)	709 (70.9%)	741 (73.2%)	738 (72.9%)	719 (70.3%)	712 (70.6%)	704 (68.0%)	704 (69.3%)	6469 (71.0%)

Netherlands	*N* = 1012	*N* = 1000	*N* = 1004	*N* = 1020	*N* = 1006	*N* = 1024	*N* = 1004	*N* = 1037	*N* = 1022	*N* = 9129
EQ-VAS score: mean (SD)	74.1 (22.4)	73.9 (22.0)	73.0 (22.5)	71.5 (24.8)	70.4 (24.3)	70.3 (25.2)	71.8 (24.5)	70.5 (25.8)	70.3 (24.6)	71.8 (24.1)
EQ-5D-5L index: mean (SD)	0.84 (0.21)	0.86 (0.18)	0.85 (0.20)	0.85 (0.20)	0.84 (0.22)	0.85 (0.21)	0.86 (0.20)	0.84 (0.22)	0.85 (0.21)	0.85 (0.21)
Mobility: any problems *N* (%)	276 (27.3%)	279 (27.9%)	290 (28.9%)	278 (27.3%)	301 (29.9%)	278 (27.1%)	284 (28.3%)	292 (28.2%)	276 (27.0%)	2554 (28.0%)
Self-care: any problems *N* (%)	107 (10.6%)	81 (8.1%)	117 (11.7%)	105 (10.3%)	125 (12.4%)	107 (10.4%)	120 (12.0%)	138 (13.3%)	119 (11.6%)	1019 (11.2%)
Usual activities: any problems *N* (%)	324 (32.0%)	311 (31.1%)	277 (27.6%)	275 (27.0%)	326 (32.4%)	307 (30.0%)	306 (30.5%)	347 (33.5%)	290 (28.4%)	2763 (30.3%)
Pain/discomfort: any problems *N* (%)	528 (52.2%)	515 (51.5%)	481 (47.9%)	498 (48.8%)	504 (50.1%)	485 (47.4%)	456 (45.4%)	518 (50.0%)	485 (47.5%)	4470 (49.0%)
Anxiety/depression: any problems *N* (%)	444 (43.9%)	372 (37.2%)	371 (37.0%)	406 (39.8%)	411 (40.9%)	390 (38.1%)	324 (32.3%)	352 (33.9%)	378 (37.0%)	3448 (37.8%)
Any dimension: any problems *N* (%)	690 (68.2%)	668 (66.8%)	638 (63.5%)	663 (65.0%)	658 (65.4%)	644 (62.9%)	593 (59.1%)	631 (60.8%)	636 (62.2%)	5821 (63.8%)

France	*N* = 1000	*N* = 1003	*N* = 1001	*N* = 1017	*N* = 1012	*N* = 1064	*N* = 1013	*N* = 1011	*N* = 1139	*N* = 9260
EQ-VAS score: mean (SD)	73.5 (21.5)	73.6 (21.0)	71.9 (22.4)	70.8 (23.4)	69.4 (22.4)	69.7 (23.2)	70.2 (23.1)	70.7 (23.6)	71.1 (22.6)	71.2 (22.6)
EQ-5D-5L index: mean (SD)	0.94 (0.10)	0.94 (0.10)	0.94 (0.09)	0.94 (0.10)	0.94 (0.10)	0.94 (0.10)	0.94 (0.10)	0.94 (0.11)	0.94 (0.11)	0.94 (0.10)
Mobility: any problems *N* (%)	212 (21.2%)	212 (21.1%)	229 (22.9%)	229 (22.5%)	240 (23.7%)	231 (21.7%)	220 (21.7%)	242 (23.9%)	275 (24.1%)	2090 (22.6%)
Self-care: any problems *N* (%)	85 (8.5%)	84 (8.4%)	101 (10.1%)	100 (9.8%)	131 (12.9%)	124 (11.7%)	124 (12.2%)	134 (13.3%)	148 (13.0%)	1031 (11.1%)
Usual activities: any problems N (%)	202 (20.2%)	194 (19.3%)	194 (19.4%)	195 (19.2%)	223 (22.0%)	206 (19.4%)	186 (18.4%)	206 (20.4%)	236 (20.7%)	1842 (19.9%)
Pain/discomfort: any problems *N* (%)	533 (53.3%)	551 (54.9%)	449 (44.9%)	473 (46.5%)	519 (51.3%)	487 (45.8%)	454 (44.8%)	470 (46.5%)	532 (46.7%)	4468 (48.3%)
Anxiety/depression: any problems *N* (%)	526 (52.6%)	476 (47.5%)	482 (48.2%)	549 (54.0%)	518 (51.2%)	538 (50.6%)	455 (44.9%)	462 (45.7%)	529 (46.4%)	4535 (49.0%)
Any dimension: any problems *N* (%)	725 (72.5%)	717 (71.5%)	656 (65.5%)	716 (70.4%)	701 (69.3%)	707 (66.4%)	640 (63.2%)	647 (64.0%)	725 (63.7%)	6234 (67.3%)

Portugal	*N* = 1064	*N* = 1015	*N* = 1001	*N* = 1015	*N* = 1005	*N* = 1032	*N* = 1001	*N* = 1045	*N* = 1036	*N* = 9214
EQ-VAS score: mean (SD)	79.4 (18.3)	80.1 (17.0)	79.3 (17.4)	79.2 (17.8)	75.8 (18.7)	77.0 (17.8)	77.9 (18.0)	78.4 (18.9)	78.0 (17.9)	78.4 (18.0)
EQ-5D-5L index: mean (SD)	0.92 (0.11)	0.92 (0.12)	0.92 (0.13)	0.92 (0.13)	0.91 (0.13)	0.92 (0.12)	0.92 (0.11)	0.93 (0.11)	0.93 (0.11)	0.92 (0.12)
Mobility: any problems *N* (%)	157 (14.8%)	170 (16.7%)	150 (15.0%)	139 (13.7%)	142 (14.1%)	147 (14.2%)	127 (12.7%)	133 (12.7%)	122 (11.8%)	1287 (14.0%)
Self-care: any problems *N* (%)	42 (3.9%)	62 (6.1%)	61 (6.1%)	62 (6.1%)	59 (5.9%)	70 (6.8%)	55 (5.5%)	62 (5.9%)	46 (4.4%)	519 (5.6%)
Usual activities: any problems *N* (%)	152 (14.3%)	176 (17.3%)	138 (13.8%)	137 (13.5%)	160 (15.9%)	150 (14.5%)	125 (12.5%)	147 (14.1%)	120 (11.6%)	1305 (14.2%)
Pain/discomfort: any problems *N* (%)	492 (46.2%)	496 (48.9%)	413 (41.3%)	401 (39.5%)	453 (45.1%)	441 (42.7%)	394 (39.4%)	395 (37.8%)	391 (37.7%)	3876 (42.1%)
Anxiety/depression: any problems *N* (%)	581 (54.6%)	507 (50.0%)	489 (48.9%)	568 (56.0%)	593 (59.0%)	583 (56.5%)	489 (48.9%)	484 (46.3%)	501 (48.4%)	4795 (52.0%)
Any dimension: any problems *N* (%)	752 (70.7%)	714 (70.3%)	650 (64.9%)	674 (66.4%)	726 (72.2%)	720 (69.8%)	630 (62.9%)	642 (61.4%)	648 (62.5%)	6156 (66.8%)

Italy	*N* = 1075	*N* = 1008	*N* = 1009	*N* = 1002	*N* = 1009	*N* = 1024	*N* = 1013	*N* = 1039	*N* = 1057	*N* = 9236
EQ-VAS score: mean (SD)	74.8 (20.1)	74.6 (20.4)	74.1 (20.8)	72.6 (21.5)	71.0 (21.3)	71.5 (20.8)	72.3 (20.5)	72.8 (21.6)	70.2 (22.8)	72.7 (21.2)
EQ-5D-5L index: mean (SD)	0.89 (0.16)	0.89 (0.15)	0.90 (0.16)	0.89 (0.16)	0.87 (0.19)	0.89 (0.17)	0.90 (0.16)	0.89 (0.16)	0.88 (0.19)	0.89 (0.17)
Mobility: any problems *N* (%)	182 (16.9%)	195 (19.3%)	179 (17.7%)	183 (18.3%)	214 (21.2%)	184 (18.0%)	201 (19.8%)	202 (19.4%)	209 (19.8%)	1749 (18.9%)
Self-care: any problems *N* (%)	93 (8.7%)	105 (10.4%)	87 (8.6%)	84 (8.4%)	123 (12.2%)	105 (10.3%)	107 (10.6%)	126 (12.1%)	142 (13.4%)	972 (10.5%)
Usual activities: any problems *N* (%)	218 (20.3%)	209 (20.7%)	146 (14.5%)	156 (15.6%)	197 (19.5%)	177 (17.3%)	174 (17.2%)	182 (17.5%)	198 (18.7%)	1657 (17.9%)
Pain/discomfort: any problems *N* (%)	581 (54.0%)	566 (56.2%)	442 (43.8%)	471 (47.0%)	528 (52.3%)	486 (47.5%)	439 (43.3%)	447 (43.0%)	477 (45.1%)	4437 (48.0%)
Anxiety/depression: any problems *N* (%)	566 (52.7%)	527 (52.3%)	449 (44.5%)	561 (56.0%)	504 (50.0%)	514 (50.2%)	449 (44.3%)	471 (45.3%)	504 (47.7%)	4545 (49.2%)
Any dimension: any problems *N* (%)	785 (73.0%)	729 (72.3%)	635 (62.9%)	713 (71.2%)	709 (70.3%)	694 (67.8%)	629 (62.1%)	647 (62.3%)	686 (64.9%)	6227 (67.4%)

Wave 1: April 2020; Wave 2: June 2020; Wave 3: September 2020; Wave 4: November 2020; Wave 5: January/February 2021; Wave 6: April 2021; Wave 7: June/July 2021; Wave 8: September 2021; Wave 9: December 2021/January 2022

On average across all nine waves, the proportion of respondents reporting any problems in at least one of the EQ-5D dimensions ranged between 63.8% (Netherlands) and 71.0% (Denmark). Variation (range) in this proportion between waves was smallest in Denmark (68.0% in wave 8 to 74.4% in wave 2) and largest in Italy (62.1% in wave 7 to 73.0% in wave 1).

Among the five EQ-5D dimensions, anxiety/depression was—on average across all nine waves—the most frequently affected dimension in Portugal (52.0%), United Kingdom (50.2%), Italy (49.2%) and France (49.0%), whereas it ranked second in Germany (41.7%), Denmark (39.3%) and the Netherlands (37.8%). Variation in the proportion of respondents reporting problems with anxiety/depression was smallest in Denmark (36.5% in wave 1 to 42.8% in wave 4) and largest in Portugal (46.3% in wave 8 to 59.0% in wave 5).

The EQ-5D dimension pain/discomfort, was—on average across all nine waves—the most frequently affected dimension in Denmark (58.3%), Germany (55.8%) and the Netherlands (49.0%), whereas it ranked second in France (48.3%), Italy (48.0%), United Kingdom (47.3%) and Portugal (42.1%). Variation in the proportion of respondents reporting problems with pain/discomfort was smallest in the United Kingdom (44.0% in wave 3 to 50.2% in wave 2) and largest in Italy (43.0% in wave 8 to 56.2% in wave 2). In all countries, the EQ-5D dimension self-care was least frequently affected on average across all nine waves, with the proportion of respondents who reported problems ranging from 5.6% in Portugal to 17.4% in the United Kingdom. Frequency of problems in the dimensions usual activities (14.2% in Portugal to 34.4% in Denmark) and mobility (14.0% in Portugal to 36.3% in Germany) ranked third or fourth among the five EQ-5D dimensions in the considered countries.

On average across all nine waves, the EQ-VAS score ranged from 70.1 in the United Kingdom to 78.4 in Portugal. Variation in the EQ-VAS score between waves was smallest in the Netherlands (70.3 in waves 6 and 9 to 74.1 in wave 1) and largest in the United Kingdom (67.9 in wave 5 to 73.1 in wave 2). Moreover, on average across all nine waves, the EQ-5D-5L index ranged from 0.82 in Denmark to 0.94 in France. Variation in the EQ-5D-5L index between waves was smallest in France (0.94 in each wave) and largest in the United Kingdom (0.81 in wave 8 to 0.85 in wave 3) and Denmark (0.80 in wave 4 and 5 to 0.84 in wave 1).

### Longitudinal regression analysis

Findings of conditional FE logistic regressions (with problems in the five EQ-5D dimensions and problems in any dimension as outcome measures) are given in Table 3. An increase in the likelihood of problems in all five EQ-5D dimensions (and problems in any dimension) was associated with an increase in the perceived risk to one’s own health from COVID-19 (e.g., with mobility as outcome measure: OR: 1.18, 95% CI: 1.11–1.26, *p* < 0.001) and the occurrence of an unconfirmed infection with the novel coronavirus (e.g., with pain/discomfort as outcome measure: OR: 1.76, 95% CI: 1.36–2.28, *p* < 0.001; except for problems with depression/anxiety). Occurrence of a confirmed infection was associated with an increased likelihood of problems in mobility, self-care and usual activities. Moreover, changes from ‘easily’ to ‘some income difficulties’ or ‘great income difficulties’ were associated with an increased likelihood of problems in all dimensions except for problems with mobility. An increase in the COVID-19 stringency index was associated with an increase in the likelihood of problems in all dimensions (e.g., with anxiety/depression as outcome measure: OR: 1.02, 95% CI: 1.01–1.02, *p* < 0.001) except for problems with mobility. Apart from that, increases in age were associated with an increased likelihood of problems in mobility, self-care and usual activities.

**Table 3:** Determinants of HRQoL (in terms of problems (0 = no problem; 1 = any problem) in the EQ-5D dimensions and problems with any dimension). Results of conditional FE logistic regressions (ECOS; wave 1 to wave 9)

Independent variables	Mobility	Self-care	Usual activities	Pain/discomfort	Anxiety/depression	Any problem
Age	1.05***	1.05***	1.03**	1.01 +	1.00	1.00
	(1.03–1.07)	(1.03–1.08)	(1.01–1.05)	(1.00–1.03)	(0.98–1.02)	(0.98–1.02)
Education:-Middle (Ref.: low education)	0.98	1.34*	0.83*	1.13	0.98	0.96
	(0.82–1.17)	(1.06–1.69)	(0.69–0.99)	(0.96–1.33)	(0.84–1.15)	(0.80–1.14)
High	1.12	1.75***	0.87	1.11	0.94	0.79*
	(0.89–1.42)	(1.30–2.34)	(0.69–1.09)	(0.89–1.37)	(0.77–1.16)	(0.64–0.99)
Professional group:-Education (Ref.: Health-related sector)	1.20	0.80	1.02	1.18	1.14	1.08
	(0.86–1.67)	(0.54–1.19)	(0.74–1.42)	(0.88–1.59)	(0.85–1.53)	(0.79–1.47)
Food retail	1.22	0.87	0.99	1.34 +	1.20	1.31
	(0.85–1.74)	(0.57–1.34)	(0.69–1.41)	(0.97–1.84)	(0.87–1.65)	(0.93–1.86)
Research	1.44 +	0.84	1.17	1.34	1.01	1.40 +
	(0.94–2.20)	(0.52–1.38)	(0.78–1.75)	(0.93–1.93)	(0.70–1.45)	(0.95–2.08)
Other	1.18	0.92	1.16	1.26*	1.03	1.10
	(0.92–1.51)	(0.67–1.25)	(0.91–1.47)	(1.01–1.57)	(0.83–1.28)	(0.87–1.39)
Income (ability to make ends meet):-With great difficulty (Ref.: easily)	1.25 +	1.74***	1.64***	1.58***	1.99***	2.05***
	(0.97–1.62)	(1.28–2.36)	(1.28–2.11)	(1.26–1.99)	(1.58–2.50)	(1.59–2.64)
With some difficulty	1.22 +	1.52**	1.39**	1.23*	1.48***	1.45***
	(1.00–1.50)	(1.18–1.95)	(1.13–1.70)	(1.04–1.46)	(1.25–1.75)	(1.22–1.72)
Fairly easily	1.07	1.26*	1.14	1.10	1.17*	1.13 +
	(0.90–1.28)	(1.00–1.57)	(0.95–1.36)	(0.96–1.27)	(1.01–1.34)	(0.98–1.30)
Infection with the novel coronavirus:-Yes, confirmed (Ref.: no)	1.59***	1.72***	1.59***	1.09	1.04	1.01
	(1.29–1.97)	(1.35–2.20)	(1.28–1.96)	(0.91–1.32)	(0.85–1.26)	(0.82–1.25)
Yes, but not yet confirmed	1.78***	1.54**	1.69***	1.76***	1.28 +	1.66***
	(1.36–2.34)	(1.14–2.08)	(1.30–2.21)	(1.36–2.28)	(1.00–1.66)	(1.24–2.22)
Don’t know	1.16 +	0.92	1.18*	1.21**	1.21**	1.33***
	(0.99–1.36)	(0.74–1.13)	(1.02–1.37)	(1.06–1.37)	(1.06–1.38)	(1.15–1.53)
Own risk of getting infected with the coronavirus (from 1 = no risk at all to 5 = very high risk)	0.97	1.07 +	1.01	1.03	1.01	1.03
	(0.91–1.04)	(0.99–1.16)	(0.95–1.07)	(0.97–1.08)	(0.96–1.07)	(0.97–1.09)
Risk to one’s own health from COVID-19 (from 1 = no risk at all to 5 = very high risk)	1.18***	1.13**	1.12***	1.13***	1.07*	1.10**
	(1.11–1.26)	(1.05–1.23)	(1.06–1.20)	(1.07–1.19)	(1.01–1.13)	(1.04–1.17)
Risk to the health of one’s own family members from COVID-19 (from 1 = no risk at all to 5 = very high risk)	0.98	0.98	0.97	1.01	1.07*	1.03
	(0.92–1.04)	(0.91–1.06)	(0.92—1.03)	(0.96–1.07)	(1.01–1.12)	(0.97–1.09)
Risk to the health of people in one’s own community from COVID-19 (from 1 = no risk at all to 5 = very high risk)	0.99	0.96	0.98	0.96	1.04	1.01
	(0.93–1.05)	(0.89–1.04)	(0.93–1.04)	(0.92–1.02)	(0.98–1.09)	(0.96–1.07)
COVID-19 stringency index (from 0 to 100, with 100 = strictest))	1.00	1.00*	1.00*	1.01***	1.02***	1.02***
	(1.00–1.00)	(0.99–1.00)	(1.00–1.01)	(1.01–1.01)	(1.01–1.02)	(1.01–1.02)
Observations	14,962	9110	15,367	21,196	21,148	19,612
Number of Individuals	3058	1886	3215	4378	4306	3974
Pseudo R^2^	0.009	0.016	0.008	0.008	0.019	0.019

Odds ratios are reported; 95% CI intervals in parentheses; ***p < 0.001, **p < 0.01, *p < 0.05, + *p* < 0.10; Listwise deletion was used to handle missing values

Findings of linear FE regressions (with EQ-VAS and EQ-5D-5L index as outcome measures) are shown in Table 4. Decreases in the EQ-VAS score were associated with increasing age (*β* = − 0.25, *p* < 0.001), emerging perceived income difficulties (e.g., from ‘easily’ to ‘great difficulty’: *β* = − 2.91, *p* < 0.001), a confirmed infection with the novel coronavirus (*β* = − 2.52, *p* < 0.001), an unconfirmed infection with the novel coronavirus (*β* = − 1.72, *p* < 0.05), increases in the perceived risk to one’s own health from COVID-19 (*β* = − 0.85, *p* < 0.001) and increases in the COVID-19 stringency index (*β* = − 0.01, *p* < 0.05). In terms of effect sizes, findings remained very similar when the EQ-5D-5L index was used (compared to the findings regarding the EQ-VAS). Please see Table 4 for further details.

**Table 4:** Determinants of HRQoL (in terms of EQ-VAS and EQ-5D-5L index). Results of linear FE regressions (ECOS; wave 1 to wave 9)

Independent variables	EQ-VAS	EQ-5D-5L index
Age	− 0.25*** (0.05)	− 0.002*** (0.000)
Education:-Middle (Ref.: low education)	− 0.47 (0.45)	− 0.000 (0.003)
High	− 0.70 (0.60)	− 0.002 (0.005)
Professional group:-Education (Ref.: Health-related sector)	− 0.20 (0.92)	− 0.003 (0.007)
Food retail	− 0.01 (1.10)	− 0.004 (0.008)
Research	− 1.10 (1.06)	− 0.009 (0.009)
Other	− 0.38 (0.69)	− 0.007 (0.005)
Income (ability to make ends meet):-With great difficulty (Ref.: easily)	− 2.91*** (0.74)	− 0.031*** (0.006)
With some difficulty	− 1.63*** (0.46)	− 0.012** (0.004)
Fairly easily	− 0.65 + (0.35)	− 0.005 + (0.003)
Infection with the novel coronavirus:-Yes, confirmed (Ref.: no)	− 2.52*** (0.65)	− 0.015** (0.005)
Yes, but not yet confirmed	− 1.72* (0.83)	− 0.014 + (0.007)
Don’t know	0.88** (0.30)	− 0.003 (0.002)
Own risk of getting infected with the coronavirus (from 1 = no risk at all to 5 = very high risk)	− 0.24 + (0.14)	− 0.002* (0.001)
Risk to one’s own health from COVID-19 (from 1 = no risk at all to 5 = very high risk)	− 0.85*** (0.15)	− 0.004*** (0.001)
Risk to the health of one’s own family members from COVID-19 (from 1 = no risk at all to 5 = very high risk)	0.03 (0.13)	− 0.001 (0.001)
Risk to the health of people in one’s own community from COVID-19 (from 1 = no risk at all to 5 = very high risk)	0.19 (0.13)	0.001 (0.001)
COVID-19 stringency index (from 0 to 100, with 100 = strictest))	− 0.01* (0.01)	− 0.000*** (0.000)
Constant	90.12*** (2.85)	1.004*** (0.024)
Observations	50,418	50,485
Number of Individuals	11,755	11,768
R^2^	0.005	0.005

Unstandardized beta-coefficients are reported; 95% confidence intervals in parentheses; ***p < 0.001, ** *p* < 0.01, *p < 0.05, +* p* < 0.10; Listwise deletion was used to handle missing values

In sensitivity analyses, we added marital status (worth repeating: quantified from wave 3 onwards) to our linear FE regression model (with EQ-VAS and EQ-5D-5L index as outcome). However, our findings remained virtually the same in terms of significance (results not shown, but available upon request). Moreover, we conducted a sensitivity analysis where we trichotomized the infection with the novel coronavirus (no; don’t know; yes, confirmed or unconfirmed). The key results remained very similar. The results are presented in Supplementary Table 3 and Supplementary Table 4.

In further sensitivity analysis, we restricted our FE regressions to individuals who participated in at least five waves. Our findings remained nearly the same. The findings are shown in Supplementary Table 5 and Supplementary Table 6.

In another sensitivity analysis, we used a FE (conditional) ordered logistic regression model [26] (based on the "blow-up and cluster" (BUC) estimator from Baetschmann et al. [27]) to examine the determinants of problems in the five EQ-5D-5L dimensions (in each case: with all five response levels). Additionally, also based on a FE (conditional) ordered logistic regression model, the determinants of a count score for problems in all five EQ-5D-5L dimensions (i.e., the count score ranges from 5 to 25, with higher values reflecting more problems in the EQ-5D-5L dimensions) were examined. The results are comparable to our main findings. These additional results are given in Supplementary Table 7.

### Drop-out analysis

In Supplementary Table 8, a drop-out analysis is shown. To this end, we compared individuals who completed all nine waves and individuals who only participated in wave 1 (in terms of sociodemographic factors and EQ-VAS). While continuous participants were significantly older, had a higher educational level, belonged to other professional groups more often, and had less perceived income difficulties compared to individuals who only participated in wave 1, no significant differences were identified regarding sex and EQ-VAS.

## Discussion

Based on longitudinal data from 9 waves during the COVID-19 pandemic, this study aimed at describing HRQoL measured by the EQ-5D-5L in seven European countries and to analyze the impact of sociodemographic and COVID-19-related variables.

On average across all waves, about two thirds of respondents reported problems in at least one of the EQ-5D dimensions, with a variation of up to approximately 10% between countries as well as between waves within countries. This proportion is at the high end of what has been reported from general population surveys that used the EQ-5D-5L before the pandemic. For example, the respective proportion was 37.4% in a study conducted 2011 in Spain (*N* = 21,007) [28], 52.5% in a study conducted 2011 in Germany (*N* = 2469) [29], 54.4% in a study conducted 2015/16 in Ireland (*N* = 1131) [30], and 69.3% in another study conducted 2015/16 in Germany (*N* = 4998) [31]. This points at decreased HRQoL during the pandemic.

Among the five EQ-5D dimensions, anxiety/depression was the most frequently affected dimension in four out of the seven countries and ranked second in the remaining three countries, with between 37.8% (Netherlands) and 52.0% (Portugal) of respondents reporting problems on average across all nine waves, and peaking in Portugal (59.0%) in Wave 5 (January/February 2021) when death rates from COVID-19 in Portugal reached its maximum [32]. While the proportion of respondents reporting problems with anxiety/depression varied markedly between countries and between waves within countries, this proportion was substantially and consistently higher than reported in the aforementioned general population surveys using the EQ-5D-5L before the pandemic, where it ranged between 16.3% in Spain [28] and 25.4% in Germany [31]. In fact, country-specific comparison of the frequency of problems with anxiety/depression in our study with previous national surveys conducted in Germany (22.7% [29], 25.4% [31]) and Denmark (19.1% [33]) point at the frequency of problems having approximately doubled during the pandemic. Furthermore, before the pandemic, general population surveys using the EQ-5D-5L [28–31, 33] or the EQ-5D-3L [34] consistently reported the highest frequency of problems in the dimension pain/discomfort, with anxiety and depression ranking mostly only third or fourth. This shows that in particular mental health related quality of life was decreased during the pandemic. This goes in line with findings of the few available studies on HRQoL during the pandemic cited above as well as with numerous studies that reported an increase in mental health symptoms during the COVID-19 pandemic [35].

Also for the other EQ-5D dimensions, the proportion of respondents reporting problems tended to be higher than in available country-specific surveys conducted before the pandemic. Taking Germany and Denmark as examples and making country-specific comparisons of average problem frequencies across all waves with the frequencies reported by Grochtdreis [31], Hinz [29], and Jensen [33], the increase in the proportion of individuals reporting problems during the pandemic was + 0.9%/ + 12.8%/ + 7.1% for mobility, + 9.1%/ + 8.0%/ + 9.9% for self-care, + 0.1%/ + 10.1%/ + 7.6% for usual activities and − 0.4%+ 10.2%/ + 9.4% for pain discomfort. Thus, although probably affecting mental health primarily, the pandemic seems to be associated with increased problems in all dimension of HRQoL.

Self-rated health measured on the EQ VAS was slightly above 70 on average across all nine waves in all countries except for Portugal where it was just below 80. Thereby EQ VAS scores in all countries (except for Portugal) were 1 to 12 points lower than respective country-specific EQ VAS scores reported in general population surveys conducted before the pandemic [31, 33, 36–38]. In Portugal, the comparatively high EQ VAS score (as well as the small frequency of problems in the EQ-5D dimensions mobility and self-care) is likely to be due to the relatively small proportion of individuals aged 65 + in all waves.

Valuation of HRQoL based on county-specific societal preferences (EQ-5D-5L index) ranged from 0.82 in Denmark to 0.94 in France. Comparable country-specific index values based on the EQ-5D-5L are only available from general population surveys conducted before the pandemic in Denmark [33] and Germany [31] which were higher by 0.08 and 0.03, respectively.

With regard to the determinants of HRQoL, FE regressions showed that in particular occurrence of infection was associated with problems in all EQ-5D dimension and a reduction in EQ VAS score as well as EQ-5D-5L index. These problems are likely to be caused by the numerous symptoms of COVID-19 [1] as well as the required isolation which may affect social and mental HRQoL. We assume that unconfirmed infections tend to be rather recent infections causing acute symptoms and/or requiring isolation—this being the reason for problems in nearly all EQ-5D dimensions. Both, the occurrence of unconfirmed and confirmed infections were significantly associated with a decrease in EQ VAS score, whereas only confirmed infections were significantly associated with a decrease in the EQ-5D-5L index. Furthermore, an increase in the perceived risk to one’s own health from COVID-19 was associated with an increased likelihood of problems in all EQ-5D dimensions, a reduced EQ VAS score and a reduced EQ-5D-5L index. This might be explained by fear of COVID-19 and avoidance of social contacts [3] in order to reduce the risk of infection. Surprisingly, changes in perceived risk to the health of one’s own family members or of people in one’s own community were not significantly associated with problems in EQ-5D dimensions nor EQ VAS score/EQ-5D-5L index (with the exception of an association between perceived risk to the health of one's own family members and problems with anxiety/depression). Furthermore, the occurrence of great income difficulties as a possible economic consequence of the pandemic was associated with an increased likelihood of problems in all EQ-5D dimension except for mobility, and a reduced EQ VAS score/EQ-5D-5L index. Not surprisingly this association was most pronounced with the EQ-5D dimension anxiety/depression. Thus, mental health stress caused by economic problems seems to also affect physical and social dimensions of HRQoL. Finally, an increase in the COVID-19 stringency index was associated with an increase in the likelihood of problems with usual activities, pain/discomfort and anxiety/depression, as well as a reduced EQ VAS score and EQ-5D index. Again, this seems plausible because this COVID-19 stringency index reflects the stringency of government measures used to reduce social contacts. By restricting usual activities, these measures were likely to also affect mental and physical health [4].

### Strengths and limitations

Some strengths are worth noting. As the first study, HRQoL in several European countries was described throughout the course of the COVID-19 pandemic (longitudinal data including nine waves from April 2020 to December 2021/January 2022). Thus, this study markedly extends our current knowledge in this research area. HRQoL was quantified using the widely used EQ-5D-5L. The determinants of HRQoL were examined using FE regressions. This choice substantially mitigates the key challenge of unobserved heterogeneity [23]. It should be acknowledged that the ECOS study focused on the general adult population in several European countries. By using an online survey, some population groups such as the oldest olds residing in institutionalized settings were less likely to participate and should be examined by future research. In Portugal, in all waves the proportion of respondents aged 65 + was markedly smaller compared to the Portuguese census population (average proportion of only 12.1% compared to 21.9% in census) which may have biased the results. Furthermore, data on morbidity (e.g., the number of health conditions an individual is living with), which is an important factor of HRQoL, was not collected in the ECOS study. Although the variable on perceived risk to one’s own health from COVID-19 might be considered a proxy, changes in morbidity may not have been sufficiently controlled for in FE regressions. Additionally, some attrition bias has been identified. However, significant differences regarding EQ-VAS were not identified between individuals who only participated in wave 1 and continuous participants. Moreover, additional FE regression analyses revealed nearly the same results when we further restricted our sample.

### Conclusion

Compared to national general population surveys conducted before the pandemic, we found large proportions of respondents reporting problems in the dimensions of HRQoL measured by the EQ-5D-5L throughout the pandemic, especially for anxiety/depression. In particular, the occurrence of infection, changes in the perceived risk to one’s own health from COVID-19, the occurrence of great income difficulties and an increase in the stringency of government response seem to be associated with impairment of HRQoL. These findings may support policy makers in maintaining HRQoL of populations when designing policies against the spread of COVID-19.

## Supplementary Information

Below is the link to the electronic supplementary material.

Supplementary file1 (DOC 462 KB)

## Data Availability

The data cannot be shared publicly because we have no permission from the respondents of our survey to share the de-identified dataset with the general public (Ethics committee: University of Hamburg). Data requests can be directed to the data access committee of our COVID-19 Survey (contact via jonas.schreyoegg@uni-hamburg.de or info@hche.de).

## Code availability

The Stata code is available upon request from André Hajek (a.hajek@uke.de).
