# Supplementary material for: Health-related quality of life in seven European countries throughout the course of the COVID-19 pandemic: evidence from the European COvid Survey (ECOS)
Source: Qual Life Res. 2023 Feb 5;32(6):1631–44. doi: 10.1007/s11136-022-03334-5 (PMC9899332; doi:10.1007/s11136-022-03334-5)
Supplement: Supplementary file 1 — Supplementary file1 (DOC 462 KB) [file 11136_2022_3334_MOESM1_ESM.doc]

Supplementary Table 1. Sample characteristics (total sample), by wave

|  | | Wave 1 | Wave 2 | Wave 3 | Wave 4 | Wave 5 | Wave 6 | Wave 7 | | --- | --- | --- | --- | --- | --- | --- | | | Wave 2 | Wave 2 | Wave 3 | Wave 4 | Wave 5 | Wave 6 | Wave 7 | | --- | --- | --- | --- | --- | --- | --- | | | Wave 3 | Wave 2 | Wave 3 | Wave 4 | Wave 5 | Wave 6 | Wave 7 | | --- | --- | --- | --- | --- | --- | --- | | | Wave 4 | Wave 2 | Wave 3 | Wave 4 | Wave 5 | Wave 6 | Wave 7 | | --- | --- | --- | --- | --- | --- | --- | | | Wave 5 | Wave 2 | Wave 3 | Wave 4 | Wave 5 | Wave 6 | Wave 7 | | --- | --- | --- | --- | --- | --- | --- | | | Wave 6 | Wave 2 | Wave 3 | Wave 4 | Wave 5 | Wave 6 | Wave 7 | | --- | --- | --- | --- | --- | --- | --- | | | Wave 7 | Wave 2 | Wave 3 | Wave 4 | Wave 5 | Wave 6 | Wave 7 | | --- | --- | --- | --- | --- | --- | --- | | Wave 8 | Wave 9 |
| --- | --- | --- | --- | --- | --- | --- | --- | --- | --- | --- | --- | --- | --- | --- | --- | --- | --- | --- | --- | --- | --- | --- | --- | --- | --- | --- | --- | --- | --- | --- | --- | --- | --- | --- | --- | --- | --- | --- | --- | --- | --- | --- | --- | --- | --- | --- | --- | --- | --- | --- | --- | --- | --- | --- | --- | --- | --- | --- |
| Age | 47.1 (16.5) | 47.6 (16.5) | 48.1 (16.3) | 47.5 (16.3) | 47.9 (16.3) | 47.9 (16.4) | 47.8 (16.4) | 47.8 (16.4) | 48.5 (16.2) |
| Gender |  |  |  |  |  |  |  |  |  |
| Male | 3450 (48.2%) | 3410 (47.9%) | 3346 (47.6%) | 3416 (48.0%) | 3415 (48.3%) | 3491 (48.5%) | 3407 (48.2%) | 3480 (48.1%) | 3505 (48.0%) |
| Female | 3710 (51.8%) | 3712 (52.1%) | 3679 (52.4%) | 3699 (52.0%) | 3653 (51.7%) | 3713 (51.5%) | 3666 (51.8%) | 3752 (51.9%) | 3795 (52.0%) |
| Educational level |  |  |  |  |  |  |  |  |  |
| Low | 2036 (28.9%) | 1619 (22.9%) | 1318 (19.4%) | 1098 (17.1%) | 1171 (16.6%) | 1169 (16.2%) | 1040 (14.7%) | 1028 (14.2%) | 981 (13.4%) |
| Middle | 3142 (44.6%) | 3237 (45.8%) | 3240 (47.7%) | 3045 (47.4%) | 2919 (41.3%) | 2968 (41.2%) | 2937 (41.5%) | 2966 (41.0%) | 2936 (40.2%) |
| High | 1874 (26.6%) | 2215 (31.3%) | 2241 (33.0%) | 2275 (35.4%) | 2978 (42.1%) | 3067 (42.6%) | 3096 (43.8%) | 3238 (44.8%) | 3383 (46.3%) |
| Professional Group |  |  |  |  |  |  |  |  |  |
| Health-related sector | 614 (8.6%) | 610 (8.6%) | 581 (8.3%) | 474 (6.7%) | 508 (7.2%) | 493 (6.8%) | 527 (7.5%) | 554 (7.7%) | 580 (7.9%) |
| Education | 500 (7.0%) | 557 (7.8%) | 509 (7.2%) | 474 (6.7%) | 428 (6.1%) | 450 (6.2%) | 485 (6.9%) | 463 (6.4%) | 528 (7.2%) |
| Food retail | 395 (5.5%) | 283 (4.0%) | 276 (3.9%) | 181 (2.5%) | 211 (3.0%) | 196 (2.7%) | 217 (3.1%) | 206 (2.8%) | 186 (2.5%) |
| Research | 185 (2.6%) | 169 (2.4%) | 173 (2.5%) | 105 (1.5%) | 119 (1.7%) | 108 (1.5%) | 132 (1.9%) | 121 (1.7%) | 125 (1.7%) |
| Other | 5466 (76.3%) | 5503 (77.3%) | 5486 (78.1%) | 5881 (82.7%) | 5802 (82.1%) | 5957 (82.7%) | 5712 (80.8%) | 5888 (81.4%) | 5881 (80.6%) |
| Perceived income difficulties |  |  |  |  |  |  |  |  |  |
| With great difficulty | 819 (11.4%) | 647 (9.1%) | 585 (8.3%) | 568 (8.0%) | 560 (7.9%) | 562 (7.8%) | 558 (7.9%) | 553 (7.6%) | 546 (7.5%) |
| With some difficulty | 2722 (38.0%) | 2615 (36.7%) | 2518 (35.8%) | 2577 (36.2%) | 2553 (36.1%) | 2449 (34.0%) | 2366 (33.5%) | 2434 (33.7%) | 2588 (35.5%) |
| Fairly easily | 2770 (38.7%) | 2892 (40.6%) | 2918 (41.5%) | 2936 (41.3%) | 2910 (41.2%) | 3082 (42.8%) | 3053 (43.2%) | 3084 (42.6%) | 3082 (42.2%) |
| Easily | 849 (11.9%) | 968 (13.6%) | 1004 (14.3%) | 1034 (14.5%) | 1045 (14.8%) | 1111 (15.4%) | 1096 (15.5%) | 1161 (16.1%) | 1084 (14.8%) |
| Infection with the novel coronavirus |  |  |  |  |  |  |  |  |  |
| Yes, confirmed | 122 (1.7%) | 126 (1.8%) | 138 (2.0%) | 227 (3.2%) | 366 (5.2%) | 462 (6.4%) | 544 (7.7%) | 631 (8.7%) | 887 (12.2%) |
| Yes, but not confirmed by tests | 188 (2.6%) | 207 (2.9%) | 133 (1.9%) | 134 (1.9%) | 154 (2.2%) | 123 (1.7%) | 216 (3.1%) | 189 (2.6%) | 194 (2.7%) |
| No | 5351 (74.7%) | 5787 (81.3%) | 6001 (85.4%) | 6127 (86.1%) | 6044 (85.5%) | 6187 (85.9%) | 6009 (85.0%) | 6062 (83.8%) | 5869 (80.4%) |
| Don’t Know | 1499 (20.9%) | 1002 (14.1%) | 753 (10.7%) | 627 (8.8%) | 504 (7.1%) | 432 (6.0%) | 304 (4.3%) | 350 (4.8%) | 350 (4.8%) |
| Own risk of getting infected with the coronavirus (from 1 = no risk at all to 5 = very high risk) | 2.9 (1.1) | 2.7 (0.9) | 3.0 (0.9) | 3.1 (0.9) | 3.0 (0.9) | 3.0 (0.9) | 2.8 (1.0) | 2.8 (1.0) | 3.1 (0.9) |
| Risk to one’s own health from COVID-19 (from 1 = no risk at all to 5 = very high risk) | 3.0 (1.2) | 2.8 (1.1) | 3.0 (1.1) | 3.1 (1.1) | 3.1 (1.1) | 3.1 (1.1) | 2.9 (1.1) | 2.9 (1.1) | 3.0 (1.1) |
| Risk to the health of one’s own family members from Covid-19 (from 1 = no risk at all to 5 = very high risk) | 3.3 (1.2) | 3.1 (1.1) | 3.3 (1.1) | 3.4 (1.1) | 3.4 (1.1) | 3.3 (1.1) | 3.1 (1.1) | 3.1 (1.1) | 3.2 (1.1) |
| Risk to the health of people in one’s own community from Covid-19 (from 1 = no risk at all to 5 = very high risk) | 3.2 (1.1) | 3.0 (1.0) | 3.1 (1.0) | 3.3 (1.0) | 3.3 (1.0) | 3.2 (1.0) | 3.1 (1.0) | 3.0 (1.0) | 3.2 (1.0) |
| COVID-19 Stringency Index (from 0 to 100. with 100 = strictest)) | 81.3 (5.6) | 64.2 (5.4) | 55.7 (7.6) | 67.1 (10.1) | 77.2 (7.7) | 72.0 (4.8) | 55.0 (9.4) | 50.4 (14.3) | 62.0 (16.1) |
| Daily new confirmed COVID-19 cases per million people (7-day rolling average) | 71.3 (46.1) | 10.9 (9.2) | 56.1 (35.9) | 372.6 (152.1) | 353.8 (365.5) | 236.4 (186.9) | 86.1 (110.8) | 149.7 (139.5) | 1800.9 (1010.0) |

Mean (SD) or N (%) are shown, as appropriate. Due to issues with labelling further options added from wave 4 on were aggregated into the “Other” category

Supplementary Table 2. Retention rates and comparison of country samples with census populations regarding age and gender, by country and wave

|  | Germany | | | | | | | | | |
| --- | --- | --- | --- | --- | --- | --- | --- | --- | --- | --- |
|  | Wave 1 | Wave 2 | Wave 3 | Wave 4 | Wave 5 | Wave 6 | Wave 7 | Wave 8 | Wave 9 | Census population |
| Age category (%) |  |  |  |  |  |  |  |  |  |  |
| …18-24 | 9.38 | 9.05 | 7.26 | 8.05 | 8.04 | 8.00 | 7.88 | 6.91 | 7.75 | 9.69 |
| …25-34 | 13.77 | 16.10 | 13.53 | 13.23 | 13.69 | 13.92 | 14.29 | 13.63 | 13.60 | 14.30 |
| …35-44 | 17.86 | 15.71 | 17.71 | 16.49 | 17.06 | 16.19 | 17.14 | 18.21 | 17.48 | 16.98 |
| …45-54 | 18.66 | 17.90 | 18.81 | 18.50 | 18.45 | 18.95 | 18.52 | 18.40 | 18.67 | 18.01 |
| …55-64 | 16.67 | 16.29 | 16.92 | 17.74 | 16.87 | 17.08 | 16.65 | 16.75 | 16.78 | 16.18 |
| …65+ | 23.65 | 24.95 | 25.77 | 25.98 | 25.89 | 25.86 | 25.52 | 26.10 | 25.72 | 24.83 |
| Proportion female (%) | 51.70 | 51.43 | 51.74 | 50.81 | 51.29 | 51.04 | 50.74 | 52.97 | 51.64 | 51.66 |
| Retention rate (%)* | n/a | 56.76 | 70.05 | 69.32 | 71.13 | 61.80 | 53.40 | 60.95 | 56.31 | n/a |
| Census source, link, year | Federal Statistical Office, <https://www.destatis.de/EN>, 2011 | | | | | | | | | |
|  | United Kingdom | | | | | | | | | |
| Age category (%) |  |  |  |  |  |  |  |  |  |  |
| …18-24 | 10.60 | 9.51 | 10.45 | 10.54 | 10.93 | 9.38 | 10.60 | 8.48 | 7.23 | 12.05 |
| …25-34 | 18.33 | 17.96 | 16.52 | 16.50 | 16.34 | 16.02 | 17.17 | 18.21 | 16.91 | 17.10 |
| …35-44 | 18.93 | 18.54 | 18.51 | 17.89 | 18.21 | 18.75 | 17.86 | 19.65 | 18.18 | 17.89 |
| …45-54 | 17.44 | 17.39 | 18.31 | 18.29 | 18.11 | 18.85 | 18.16 | 18.59 | 18.77 | 17.72 |
| …55-64 | 15.16 | 16.04 | 15.82 | 15.61 | 15.55 | 15.72 | 15.51 | 14.93 | 17.01 | 15.05 |
| …65+ | 19.52 | 20.56 | 20.40 | 21.17 | 20.87 | 21.29 | 20.71 | 20.13 | 21.90 | 20.19 |
| Proportion female (%) | 52.23 | 53.12 | 50.45 | 50.50 | 50.89 | 50.59 | 51.52 | 51.73 | 51.52 | 51.38 |
| Retention rate (%)* | n/a | 58.69 | 61.09 | 61.83 | 64.47 | 55.86 | 53.09 | 50.87 | 44.48 | n/a |
| Census source, link, year | Office of National Statistics (ONS), <https://www.ons.gov.uk/>, 2011 | | | | | | | | | |
|  | Denmark | | | | | | | | | |
| Age category (%) |  |  |  |  |  |  |  |  |  |  |
| …18-24 | 10.90 | 9.77 | 8.10 | 10.47 | 8.79 | 14.78 | 9.03 | 9.57 | 7.87 | 11.18 |
| …25-34 | 14.90 | 13.26 | 12.60 | 14.23 | 14.03 | 21.39 | 14.88 | 15.36 | 14.27 | 14.87 |
| …35-44 | 17.40 | 16.25 | 16.40 | 17.49 | 17.98 | 21.75 | 17.66 | 16.91 | 19.39 | 17.84 |
| …45-54 | 18.50 | 17.85 | 19.00 | 18.48 | 19.07 | 25.77 | 18.65 | 19.03 | 18.60 | 18.02 |
| …55-64 | 16.10 | 18.74 | 19.30 | 16.70 | 17.29 | 15.37 | 16.67 | 16.71 | 17.03 | 16.11 |
| …65+ | 22.20 | 24.13 | 24.60 | 22.63 | 22.83 | 0.95 | 23.12 | 22.42 | 22.83 | 21.98 |
| Proportion female (%) | 51.10 | 52.04 | 54.20 | 51.88 | 52.27 | 50.24 | 52.28 | 50.72 | 53.64 | 50.87 |
| Retention rate (%)* | n/a | 45.67 | 59.50 | 50.49 | 51.19 | 39.69 | 48.41 | 47.83 | 46.26 | n/a |
| Census source, link, year | Statistics Denmark, <https://www.dst.dk/en>. 2017 | | | | | | | | | |
|  | Netherlands | | | | | | | | | |
| Age category (%) |  |  |  |  |  |  |  |  |  |  |
| …18-24 | 10.38 | 10.20 | 9.96 | 9.41 | 10.14 | 9.08 | 9.16 | 8.78 | 7.53 | 10.84 |
| …25-34 | 15.71 | 14.50 | 15.44 | 13.63 | 13.62 | 14.45 | 14.94 | 14.37 | 14.29 | 15.18 |
| …35-44 | 18.77 | 18.60 | 18.03 | 18.92 | 19.18 | 19.43 | 18.63 | 18.61 | 18.10 | 18.82 |
| …45-54 | 18.48 | 18.90 | 18.33 | 19.51 | 19.28 | 19.43 | 19.42 | 19.58 | 20.25 | 18.73 |
| …55-64 | 16.40 | 16.80 | 17.23 | 17.35 | 16.90 | 16.89 | 16.83 | 17.84 | 18.10 | 16.35 |
| …65+ | 20.26 | 21.00 | 21.02 | 21.18 | 20.87 | 20.70 | 21.02 | 20.83 | 21.72 | 20.10 |
| Proportion female (%) | 50.99 | 51.00 | 51.69 | 52.75 | 51.99 | 52.05 | 52.09 | 52.75 | 53.82 | 50.99 |
| Retention rate (%)* | n/a | 56.20 | 52.79 | 56.67 | 63.42 | 65.14 | 61.06 | 56.32 | 53.72 | n/a |

| Census source, link, year | Centraal Bureau voor de Statistiek (CBS), <https://www.cbs.nl/>, 2010 | | | | | | | | | |
| --- | --- | --- | --- | --- | --- | --- | --- | --- | --- | --- |
|  | France | | | | | | | | | |
| Age category (%) |  |  |  |  |  |  |  |  |  |  |
| …18-24 | 11.50 | 9.57 | 9.19 | 10.72 | 9.49 | 9.02 | 9.67 | 10.78 | 6.76 | 11.36 |
| …25-34 | 16.10 | 16.55 | 15.28 | 15.93 | 15.42 | 16.92 | 15.89 | 15.03 | 15.19 | 16.18 |
| …35-44 | 18.20 | 18.25 | 18.88 | 18.49 | 18.68 | 18.33 | 18.66 | 18.20 | 19.14 | 18.07 |
| …45-54 | 17.20 | 17.45 | 18.08 | 17.21 | 18.28 | 17.86 | 17.87 | 17.31 | 18.88 | 17.49 |
| …55-64 | 15.70 | 15.75 | 16.38 | 15.73 | 15.91 | 15.70 | 15.70 | 16.02 | 18.26 | 15.46 |
| …65+ | 21.30 | 22.43 | 22.18 | 21.93 | 22.23 | 22.18 | 22.21 | 22.65 | 21.77 | 21.45 |
| Proportion female (%) | 52.30 | 52.84 | 53.85 | 52.9 | 52.96 | 54.7 | 52.52 | 52.52 | 52.41 | 52.36 |
| Retention rate (%)* | n/a | 58.82 | 64.84 | 69.91 | 66.4 | 65.41 | 65.05 | 61.33 | 50.31 | n/a |
| Census source, link, year | National Institute of Statistics and Economic Studies (INSEE), <https://www.insee.fr/en/accueil>, 2011 | | | | | | | | | |
|  | Portugal | | | | | | | | | |
| Age category (%) |  |  |  |  |  |  |  |  |  |  |
| …18-24 | 11.56 | 10.54 | 10.69 | 13.20 | 10.65 | 10.76 | 10.89 | 11.00 | 8.69 | 9.69 |
| …25-34 | 21.15 | 19.90 | 20.98 | 22.46 | 19.90 | 21.90 | 21.58 | 22.30 | 19.59 | 18.19 |
| …35-44 | 21.52 | 20.89 | 21.08 | 23.65 | 24.28 | 22.00 | 22.38 | 19.90 | 21.04 | 18.50 |
| …45-54 | 18.14 | 18.92 | 18.38 | 20.69 | 19.50 | 19.67 | 19.48 | 19.14 | 18.82 | 17.13 |
| …55-64 | 13.25 | 15.67 | 14.59 | 14.09 | 16.62 | 13.95 | 15.78 | 14.45 | 15.35 | 14.59 |
| …65+ | 14.38 | 14.09 | 14.29 | 5.91 | 9.05 | 11.72 | 9.89 | 13.21 | 16.51 | 21.90 |
| Proportion female (%) | 52.54 | 52.41 | 52.05 | 52.61 | 50.15 | 50.48 | 51.35 | 51.87 | 49.23 | 52.24 |
| Retention rate (%)* | n/a | 46.90 | 52.95 | 39.01 | 45.47 | 39.05 | 38.26 | 49.67 | 50.48 | n/a |
| Census source, link, year | Instituto Nacional De Estatistica (INE) / Statistics Portugal, https://www.ine.pt/xportal/xmain?xpgid=ine_main&xpid=INE, 2011 | | | | | | | | | |
|  | Italy | | | | | | | | | |
| Age category (%) |  |  |  |  |  |  |  |  |  |  |
| …18-24 | 8.05 | 9.03 | 7.93 | 7.19 | 7.93 | 7.52 | 8.39 | 8.47 | 8.14 | 8.64 |
| …25-34 | 14.97 | 15.18 | 14.87 | 14.77 | 14.37 | 14.94 | 14.61 | 16.65 | 17.88 | 14.95 |
| …35-44 | 19.78 | 19.44 | 20.32 | 19.26 | 19.52 | 20.12 | 19.35 | 16.94 | 20.62 | 19.30 |
| …45-54 | 19.02 | 17.56 | 18.24 | 18.36 | 18.33 | 17.97 | 17.97 | 19.25 | 18.16 | 17.72 |
| …55-64 | 15.35 | 14.78 | 14.77 | 15.37 | 15.36 | 15.53 | 15.20 | 15.88 | 15.99 | 14.99 |
| …65+ | 22.83 | 24.01 | 23.89 | 25.05 | 24.48 | 23.93 | 24.48 | 22.81 | 19.21 | 24.41 |
| Proportion female (%) | 51.11 | 51.98 | 52.63 | 52.5 | 52.23 | 51.56 | 52.32 | 50.63 | 51.66 | 52.08 |
| Retention rate (%)* | n/a | 68.55 | 61.74 | 63.17 | 64.12 | 57.91 | 56.47 | 55.82 | 52.70 | n/a |
| Census source, link, year | Italian National Institute of Statistics, <https://www.istat.it/en/>, 2011 | | | | | | | | | |

*Retention rate is defined as the share of participants in the current wave that also participated in the wave before, e.g., share of participants in wave 4 that participated in wave 3 as well.

Supplementary Table 3. Determinants of HRQoL (in terms of problems (0 = no problem; 1 = any problem) in the EQ-5D dimensions and problems with any dimension). Results of conditional FE logistic regressions (ECOS; wave 1 to wave 9) – with three groups for infection with the novel coronavirus

| Independent variables | Mobility | Self-care | Usual activities | Pain/discomfort | Anxiety/depression | Any problem |
| --- | --- | --- | --- | --- | --- | --- |
|  |  |  |  |  |  |  |
| Age | 1.05*** | 1.05*** | 1.03** | 1.01 | 1.00 | 1.00 |
|  | (1.03 - 1.07) | (1.03 - 1.08) | (1.01 - 1.05) | (1.00 - 1.03) | (0.98 - 1.02) | (0.98 - 1.02) |
| Education: - Middle (Ref.: low education) | 0.98 | 1.34* | 0.82* | 1.12 | 0.98 | 0.95 |
|  | (0.82 - 1.17) | (1.06 - 1.69) | (0.69 - 0.99) | (0.95 - 1.33) | (0.83 - 1.15) | (0.80 - 1.13) |
| - High | 1.12 | 1.75*** | 0.87 | 1.10 | 0.94 | 0.79* |
|  | (0.89 - 1.42) | (1.31 - 2.34) | (0.69 - 1.09) | (0.89 - 1.35) | (0.76 - 1.16) | (0.63 - 0.98) |
| Professional group: - Education (Ref.: Health-related sector) | 1.20 | 0.80 | 1.02 | 1.17 | 1.14 | 1.07 |
|  | (0.86 - 1.67) | (0.54 - 1.19) | (0.74 - 1.42) | (0.87 - 1.57) | (0.85 - 1.52) | (0.78 - 1.46) |
| - Food retail | 1.22 | 0.87 | 0.99 | 1.33+ | 1.20 | 1.31 |
|  | (0.86 - 1.74) | (0.57 - 1.33) | (0.69 - 1.41) | (0.97 - 1.83) | (0.87 - 1.65) | (0.93 - 1.85) |
| - Research | 1.44+ | 0.84 | 1.17 | 1.34 | 1.00 | 1.39 |
|  | (0.94 - 2.20) | (0.52 - 1.38) | (0.78 - 1.75) | (0.93 - 1.92) | (0.69 - 1.45) | (0.94 - 2.05) |
| - Other | 1.18 | 0.92 | 1.16 | 1.25+ | 1.03 | 1.09 |
|  | (0.92 - 1.51) | (0.68 - 1.25) | (0.91 - 1.47) | (1.00 - 1.56) | (0.83 - 1.28) | (0.87 - 1.37) |
| Income (ability to make ends meet): - With great difficulty (Ref.: easily) | 1.25+ | 1.74*** | 1.64*** | 1.58*** | 1.99*** | 2.04*** |
|  | (0.97 - 1.62) | (1.28 - 2.35) | (1.28 - 2.11) | (1.26 - 1.99) | (1.58 - 2.50) | (1.58 - 2.63) |
| - With some difficulty | 1.22+ | 1.51** | 1.39** | 1.24* | 1.48*** | 1.46*** |
|  | (1.00 - 1.50) | (1.17 - 1.95) | (1.13 - 1.70) | (1.04 - 1.47) | (1.25 - 1.75) | (1.23 - 1.73) |
| - Fairly easily | 1.07 | 1.25* | 1.14 | 1.11 | 1.17* | 1.13+ |
|  | (0.90 - 1.28) | (1.00 - 1.57) | (0.95 - 1.36) | (0.96 - 1.28) | (1.01 - 1.35) | (0.99 - 1.30) |
| Infection with the novel coronavirus: - Yes, confirmed or unconfirmed (Ref.: no) | 1.66*** | 1.65*** | 1.62*** | 1.27** | 1.12 | 1.19+ |
|  | (1.38 - 1.99) | (1.34 - 2.03) | (1.36 - 1.94) | (1.08 - 1.50) | (0.95 - 1.32) | (1.00 - 1.42) |
| - Don’t know | 1.16+ | 0.92 | 1.18* | 1.20** | 1.20** | 1.32*** |
|  | (0.99 - 1.36) | (0.75 - 1.13) | (1.02 - 1.37) | (1.05 - 1.36) | (1.06 - 1.37) | (1.15 - 1.52) |
| Own risk of getting infected with the coronavirus (from 1 = no risk at all to 5 = very high risk) | 0.97 | 1.07+ | 1.01 | 1.03 | 1.01 | 1.03 |
|  | (0.91 - 1.04) | (0.99 - 1.16) | (0.95 - 1.07) | (0.97 - 1.08) | (0.96 - 1.07) | (0.97 - 1.09) |
| Risk to one’s own health from COVID-19 (from 1 = no risk at all to 5 = very high risk) | 1.18*** | 1.13** | 1.12*** | 1.13*** | 1.07* | 1.10** |
|  | (1.11 - 1.26) | (1.05 - 1.23) | (1.06 - 1.20) | (1.07 - 1.19) | (1.01 - 1.13) | (1.04 - 1.16) |
| Risk to the health of one’s own family members from COVID-19 (from 1 = no risk at all to 5 = very high risk) | 0.98 | 0.98 | 0.97 | 1.01 | 1.07* | 1.03 |
|  | (0.92 - 1.04) | (0.91 - 1.06) | (0.92 - 1.03) | (0.96 - 1.07) | (1.01 - 1.12) | (0.97 - 1.09) |
| Risk to the health of people in one’s own community from COVID-19 (from 1 = no risk at all to 5 = very high risk) | 0.98 | 0.96 | 0.98 | 0.97 | 1.04 | 1.01 |
|  | (0.93 - 1.05) | (0.89 - 1.04) | (0.93 - 1.04) | (0.92 - 1.02) | (0.98 - 1.09) | (0.96 - 1.07) |
| COVID-19 Stringency Index (from 0 to 100, with 100 = strictest)) | 1.00 | 1.00* | 1.00* | 1.01*** | 1.02*** | 1.02*** |
|  | (1.00 - 1.00) | (0.99 - 1.00) | (1.00 - 1.01) | (1.01 - 1.01) | (1.01 - 1.02) | (1.01 - 1.02) |
|  |  |  |  |  |  |  |
| Observations | 14,962 | 9,110 | 15,367 | 21,196 | 21,148 | 19,612 |
| Number of Individuals | 3,058 | 1,886 | 3,215 | 4,378 | 4,306 | 3,974 |
| Pseudo R² | 0.009 | 0.015 | 0.008 | 0.008 | 0.019 | 0.019 |

Odds Ratios are reported; 95% CI intervals in parentheses; *** p<0.001, ** p<0.01, * p<0.05, + p<0.10; Listwise deletion was used to handle missing values.

Supplementary Table 4. Determinants of HRQoL (in terms of EQ-VAS and EQ-5D-5L index). Results of linear FE regressions (ECOS; wave 1 to wave 9) – with three groups for infection with the novel coronavirus

| Independent variables | EQ-VAS | EQ-5D-5L index |
| --- | --- | --- |
|  |  |  |
| Age | -0.25*** | -0.002*** |
|  | (0.05) | (0.000) |
| Education: - Middle (Ref.: low education) | -0.48 | -0.000 |
|  | (0.45) | (0.003) |
| - High | -0.71 | -0.002 |
|  | (0.60) | (0.005) |
| Professional group: - Education (Ref.: Health-related sector) | -0.20 | -0.003 |
|  | (0.92) | (0.007) |
| - Food retail | -0.01 | -0.004 |
|  | (1.10) | (0.008) |
| - Research | -1.10 | -0.009 |
|  | (1.06) | (0.009) |
| - Other | -0.39 | -0.007 |
|  | (0.69) | (0.005) |
| Income (ability to make ends meet): - With great difficulty (Ref.: easily) | -2.91*** | -0.031*** |
|  | (0.74) | (0.006) |
| - With some difficulty | -1.62*** | -0.012** |
|  | (0.46) | (0.004) |
| - Fairly easily | -0.65+ | -0.005+ |
|  | (0.35) | (0.003) |
| Infection with the novel coronavirus: - Yes, confirmed or unconfirmed (Ref.: no) | 0.87** | -0.003 |
|  | (0.30) | (0.002) |
| - Don’t know | -2.25*** | -0.015*** |
|  | (0.54) | (0.004) |
| Own risk of getting infected with the coronavirus (from 1 = no risk at all to 5 = very high risk) | -0.24+ | -0.002* |
|  | (0.14) | (0.001) |
| Risk to one’s own health from COVID-19 (from 1 = no risk at all to 5 = very high risk) | -0.85*** | -0.004*** |
|  | (0.15) | (0.001) |
| Risk to the health of one’s own family members from COVID-19 (from 1 = no risk at all to 5 = very high risk) | 0.03 | -0.001 |
|  | (0.13) | (0.001) |
| Risk to the health of people in one’s own community from COVID-19 (from 1 = no risk at all to 5 = very high risk) | 0.19 | 0.001 |
|  | (0.13) | (0.001) |
| COVID-19 Stringency Index (from 0 to 100, with 100 = strictest)) | -0.01* | -0.000*** |
|  | (0.01) | (0.000) |
| Constant | 90.35*** | 1.004*** |
|  | (2.76) | (0.024) |
|  |  |  |
| Observations | 50,418 | 50,485 |
| Number of Individuals | 11,755 | 11,768 |
| R² | 0.005 | 0.005 |

Unstandardized beta-coefficients are reported; 95% confidence intervals in parentheses; *** p<0.001, ** p<0.01, * p<0.05, + p<0.10; Listwise deletion was used to handle missing values.

Supplementary Table 5. Determinants of HRQoL (in terms of problems (0 = no problem; 1 = any problem) in the EQ-5D dimensions and problems with any dimension). Results of conditional FE logistic regressions (ECOS; wave 1 to wave 9) – restricted to individuals who participated in at least five waves

| Independent variables | Mobility | Self-care | Usual activities | Pain/discomfort | Anxiety/depression | Any problem |
| --- | --- | --- | --- | --- | --- | --- |
|  |  |  |  |  |  |  |
| Age | 1.04*** | 1.04* | 1.02+ | 1.01 | 1.00 | 1.00 |
|  | (1.02 - 1.07) | (1.01 - 1.07) | (1.00 - 1.04) | (0.99 - 1.03) | (0.98 - 1.01) | (0.98 - 1.02) |
| Education: - Middle (Ref.: low education) | 0.99 | 1.51** | 0.83+ | 1.20+ | 0.95 | 1.00 |
|  | (0.80 - 1.21) | (1.14 - 1.99) | (0.67 - 1.02) | (0.99 - 1.45) | (0.79 - 1.15) | (0.82 - 1.22) |
| - High | 1.20 | 2.18*** | 0.91 | 1.10 | 0.88 | 0.85 |
|  | (0.90 - 1.58) | (1.52 - 3.14) | (0.68 - 1.20) | (0.86 - 1.42) | (0.69 - 1.13) | (0.65 - 1.10) |
| Professional group: - Education (Ref.: Health-related sector) | 1.04 | 0.49* | 0.86 | 1.09 | 1.15 | 1.04 |
|  | (0.65 - 1.65) | (0.26 - 0.90) | (0.54 - 1.37) | (0.72 - 1.64) | (0.78 - 1.69) | (0.69 - 1.57) |
| - Food retail | 1.08 | 0.65 | 0.78 | 1.36 | 1.23 | 1.42 |
|  | (0.68 - 1.71) | (0.36 - 1.18) | (0.48 - 1.26) | (0.89 - 2.08) | (0.81 - 1.87) | (0.90 - 2.24) |
| - Research | 1.49 | 0.70 | 1.03 | 1.57+ | 1.04 | 1.63+ |
|  | (0.86 - 2.57) | (0.35 - 1.39) | (0.59 - 1.79) | (0.96 - 2.57) | (0.64 - 1.69) | (0.99 - 2.68) |
| - Other | 1.08 | 0.82 | 1.10 | 1.39* | 1.11 | 1.19 |
|  | (0.78 - 1.50) | (0.52 - 1.29) | (0.79 - 1.54) | (1.03 - 1.87) | (0.83 - 1.48) | (0.87 - 1.62) |
| Income (ability to make ends meet): - With great difficulty (Ref.: easily) | 1.20 | 1.71** | 1.67** | 1.81*** | 1.98*** | 1.79*** |
|  | (0.86 - 1.67) | (1.15 - 2.57) | (1.19 - 2.34) | (1.34 - 2.45) | (1.47 - 2.66) | (1.29 - 2.48) |
| - With some difficulty | 1.14 | 1.32 | 1.35* | 1.26* | 1.48*** | 1.40** |
|  | (0.88 - 1.47) | (0.95 - 1.83) | (1.03 - 1.75) | (1.02 - 1.57) | (1.20 - 1.83) | (1.13 - 1.73) |
| - Fairly easily | 1.05 | 1.13 | 1.14 | 1.09 | 1.22* | 1.13 |
|  | (0.84 - 1.31) | (0.85 - 1.51) | (0.90 - 1.43) | (0.92 - 1.30) | (1.03 - 1.46) | (0.95 - 1.33) |
| Infection with the novel coronavirus: - Yes, confirmed (Ref.: no) | 1.42* | 1.42* | 1.37* | 0.86 | 0.86 | 0.77+ |
|  | (1.08 - 1.87) | (1.03 - 1.94) | (1.04 - 1.81) | (0.67 - 1.10) | (0.67 - 1.10) | (0.60 - 1.00) |
| - Yes, but not yet confirmed | 1.88*** | 1.45+ | 1.56* | 1.76** | 1.33+ | 1.63** |
|  | (1.32 - 2.68) | (0.98 - 2.14) | (1.11 - 2.20) | (1.25 - 2.47) | (0.96 - 1.84) | (1.13 - 2.34) |
| - Don’t know | 1.16 | 0.97 | 1.11 | 1.13 | 1.21* | 1.18+ |
|  | (0.95 - 1.41) | (0.75 - 1.25) | (0.92 - 1.34) | (0.96 - 1.32) | (1.03 - 1.42) | (0.99 - 1.40) |
| Own risk of getting infected with the coronavirus (from 1 = no risk at all to 5 = very high risk) | 1.01 | 1.07 | 1.00 | 1.03 | 1.01 | 1.03 |
|  | (0.93 - 1.09) | (0.97 - 1.19) | (0.92 - 1.08) | (0.96 - 1.10) | (0.94 - 1.08) | (0.96 - 1.11) |
| Risk to one’s own health from COVID-19 (from 1 = no risk at all to 5 = very high risk) | 1.19*** | 1.15** | 1.13** | 1.11** | 1.08* | 1.09* |
|  | (1.10 - 1.29) | (1.04 - 1.27) | (1.04 - 1.22) | (1.03 - 1.19) | (1.01 - 1.15) | (1.01 - 1.17) |
| Risk to the health of one’s own family members from COVID-19 (from 1 = no risk at all to 5 = very high risk) | 0.96 | 0.95 | 0.95 | 1.00 | 1.06 | 1.01 |
|  | (0.89 - 1.03) | (0.86 - 1.05) | (0.88 - 1.03) | (0.94 - 1.07) | (0.99 - 1.13) | (0.94 - 1.08) |
| Risk to the health of people in one’s own community from COVID-19 (from 1 = no risk at all to 5 = very high risk) | 0.97 | 0.99 | 1.00 | 0.95+ | 1.04 | 1.01 |
|  | (0.90 - 1.05) | (0.90 - 1.09) | (0.93 - 1.08) | (0.89 - 1.01) | (0.97 - 1.11) | (0.94 - 1.08) |
| COVID-19 Stringency Index (from 0 to 100, with 100 = strictest)) | 1.00 | 1.00 | 1.00 | 1.01*** | 1.02*** | 1.02*** |
|  | (1.00 - 1.01) | (0.99 - 1.00) | (1.00 - 1.00) | (1.01 - 1.01) | (1.01 - 1.02) | (1.01 - 1.02) |
|  |  |  |  |  |  |  |
| Observations | 10,874 | 6,476 | 10,847 | 15,057 | 15,340 | 14,272 |
| Number of Individuals | 1,618 | 957 | 1,608 | 2,230 | 2,279 | 2,113 |
| Pseudo R² | 0.008 | 0.013 | 0.006 | 0.008 | 0.018 | 0.018 |

Odds Ratios are reported; 95% CI intervals in parentheses; *** p<0.001, ** p<0.01, * p<0.05, + p<0.10; Listwise deletion was used to handle missing values.

Supplementary Table 6. Determinants of HRQoL (in terms of EQ-VAS and EQ-5D-5L index). Results of linear FE regressions (ECOS; wave 1 to wave 9) – restricted to individuals who participated in at least five waves

| Independent variables | EQ-VAS | EQ-5D-5L index |
| --- | --- | --- |
|  |  |  |
| Age | -0.27*** | -0.002** |
|  | (0.06) | (0.001) |
| Education: - Middle (Ref.: low education) | -0.50 | -0.004 |
|  | (0.50) | (0.004) |
| - High | -0.75 | -0.006 |
|  | (0.69) | (0.005) |
| Professional group: - Education (Ref.: Health-related sector) | 0.02 | 0.003 |
|  | (1.15) | (0.008) |
| - Food retail | 0.95 | 0.009 |
|  | (1.41) | (0.010) |
| - Research | -0.13 | -0.005 |
|  | (1.30) | (0.010) |
| - Other | 0.02 | -0.004 |
|  | (0.88) | (0.006) |
| Income (ability to make ends meet): - With great difficulty (Ref.: easily) | -3.46*** | -0.031*** |
|  | (0.90) | (0.007) |
| - With some difficulty | -1.54** | -0.009* |
|  | (0.55) | (0.004) |
| - Fairly easily | -0.55 | -0.004 |
|  | (0.41) | (0.003) |
| Infection with the novel coronavirus: - Yes, confirmed (Ref.: no) | -2.29** | -0.010+ |
|  | (0.80) | (0.006) |
| - Yes, unconfirmed | -1.81+ | -0.018* |
|  | (1.02) | (0.009) |
| - Don’t know | 0.96** | -0.003 |
|  | (0.36) | (0.003) |
| Own risk of getting infected with the coronavirus (from 1 = no risk at all to 5 = very high risk) | -0.04 | -0.000 |
|  | (0.16) | (0.001) |
| Risk to one’s own health from COVID-19 (from 1 = no risk at all to 5 = very high risk) | -0.80*** | -0.004** |
|  | (0.18) | (0.001) |
| Risk to the health of one’s own family members from COVID-19 (from 1 = no risk at all to 5 = very high risk) | -0.01 | -0.002 |
|  | (0.16) | (0.001) |
| Risk to the health of people in one’s own community from COVID-19 (from 1 = no risk at all to 5 = very high risk) | 0.20 | 0.001 |
|  | (0.15) | (0.001) |
| COVID-19 Stringency Index (from 0 to 100, with 100 = strictest)) | -0.01+ | -0.000*** |
|  | (0.01) | (0.000) |
| Constant | 91.02*** | 1.005*** |
|  | (3.61) | (0.032) |
|  |  |  |
| Observations | 31,636 | 31,667 |
| Number of Individuals | 4,762 | 4,762 |
| R² | 0.005 | 0.005 |

Unstandardized beta-coefficients are reported; 95% confidence intervals in parentheses; *** p<0.001, ** p<0.01, * p<0.05, + p<0.10; Listwise deletion was used to handle missing values.

Supplementary Table 7. Determinants of HRQoL in the EQ-5D dimensions (in each case: with all five response levels) and a count score for problems in all five EQ-5D-5L dimensions. Results of FE (conditional) ordered logistic regression model (ECOS; wave 1 to wave 9)

| Independent variables | Mobility | Self-care | Usual activities | Pain/discomfort | Anxiety/depression | Count score for problems in all five EQ-5D-5L dimensions |
| --- | --- | --- | --- | --- | --- | --- |
|  |  |  |  |  |  |  |
| Age | 1.04*** | 1.05*** | 1.04*** | 1.02*** | 1.01 | 1.04*** |
|  | (1.02 - 1.06) | (1.02 - 1.08) | (1.02 - 1.06) | (1.01 - 1.04) | (1.00 - 1.02) | (1.02 - 1.05) |
| Education: - Middle (Ref.: low education) | 0.98 | 1.20 | 0.89 | 1.04 | 1.07 | 1.03 |
|  | (0.83 - 1.16) | (0.94 - 1.53) | (0.75 - 1.06) | (0.90 - 1.20) | (0.92 - 1.23) | (0.90 - 1.18) |
| - High | 1.05 | 1.48** | 0.93 | 1.06 | 1.07 | 1.10 |
|  | (0.84 - 1.31) | (1.10 - 1.99) | (0.74 - 1.17) | (0.88 - 1.28) | (0.88 - 1.29) | (0.91 - 1.32) |
| Professional group: - Education (Ref.: Health-related sector) | 1.28 | 0.86 | 1.20 | 1.16 | 0.86 | 1.06 |
|  | (0.92 - 1.77) | (0.58 - 1.28) | (0.88 - 1.64) | (0.90 - 1.51) | (0.68 - 1.10) | (0.81 - 1.37) |
| - Food retail | 1.40+ | 1.22 | 1.17 | 1.19 | 1.02 | 1.22 |
|  | (1.00 - 1.95) | (0.82 - 1.82) | (0.83 - 1.63) | (0.89 - 1.59) | (0.77 - 1.34) | (0.93 - 1.60) |
| - Research | 1.54* | 1.00 | 1.33 | 1.15 | 1.01 | 1.20 |
|  | (1.05 - 2.27) | (0.65 - 1.55) | (0.90 - 1.98) | (0.84 - 1.56) | (0.75 - 1.36) | (0.90 - 1.62) |
| - Other | 1.35* | 1.14 | 1.19 | 1.17 | 1.00 | 1.17+ |
|  | (1.06 - 1.73) | (0.84 - 1.54) | (0.93 - 1.50) | (0.95 - 1.42) | (0.83 - 1.20) | (0.97 - 1.42) |
| Income (ability to make ends meet): - With great difficulty (Ref.: easily) | 1.19 | 1.44* | 1.63*** | 1.63*** | 1.92*** | 1.74*** |
|  | (0.94 - 1.50) | (1.06 - 1.96) | (1.29 - 2.05) | (1.33 - 1.99) | (1.57 - 2.36) | (1.43 - 2.11) |
| - With some difficulty | 1.11 | 1.41* | 1.32** | 1.24** | 1.39*** | 1.35*** |
|  | (0.91 - 1.34) | (1.08 - 1.85) | (1.09 - 1.60) | (1.06 - 1.46) | (1.19 - 1.62) | (1.15 - 1.57) |
| - Fairly easily | 1.02 | 1.23+ | 1.12 | 1.11 | 1.14+ | 1.13+ |
|  | (0.87 - 1.20) | (0.97 - 1.55) | (0.95 - 1.32) | (0.97 - 1.26) | (1.00 - 1.30) | (1.00 - 1.29) |
| Infection with the novel coronavirus: - Yes, confirmed (Ref.: no) | 1.37** | 1.55*** | 1.49*** | 1.23* | 1.11 | 1.41*** |
|  | (1.12 - 1.67) | (1.24 - 1.95) | (1.21 - 1.83) | (1.04 - 1.46) | (0.93 - 1.32) | (1.19 - 1.67) |
| - Yes, but not yet confirmed | 1.35* | 1.29+ | 1.22+ | 1.32** | 1.24* | 1.36** |
|  | (1.06 - 1.73) | (1.00 - 1.67) | (0.97 - 1.52) | (1.09 - 1.61) | (1.02 - 1.52) | (1.11 - 1.66) |
| - Don’t know | 1.08 | 0.98 | 1.16* | 1.11+ | 1.07 | 1.12* |
|  | (0.93 - 1.25) | (0.79 - 1.21) | (1.00 - 1.34) | (0.99 - 1.23) | (0.96 - 1.20) | (1.00 - 1.24) |
| Own risk of getting infected with the coronavirus (from 1 = no risk at all to 5 = very high risk) | 1.02 | 1.06 | 1.01 | 1.01 | 1.04 | 1.03 |
|  | (0.96 - 1.08) | (0.98 - 1.15) | (0.96 - 1.07) | (0.96 - 1.05) | (0.99 - 1.08) | (0.98 - 1.07) |
| Risk to one’s own health from COVID-19 (from 1 = no risk at all to 5 = very high risk) | 1.15*** | 1.11** | 1.09** | 1.12*** | 1.05* | 1.13*** |
|  | (1.09 - 1.22) | (1.03 - 1.20) | (1.03 - 1.16) | (1.07 - 1.17) | (1.00 - 1.10) | (1.08 - 1.18) |
| Risk to the health of one’s own family members from COVID-19 (from 1 = no risk at all to 5 = very high risk) | 1.00 | 1.02 | 1.00 | 1.00 | 1.06** | 1.02 |
|  | (0.94 - 1.06) | (0.95 - 1.09) | (0.94 - 1.05) | (0.96 - 1.05) | (1.02 - 1.11) | (0.98 - 1.06) |
| Risk to the health of people in one’s own community from COVID-19 (from 1 = no risk at all to 5 = very high risk) | 0.98 | 0.95 | 0.97 | 0.99 | 1.01 | 0.98 |
|  | (0.93 - 1.03) | (0.89 - 1.02) | (0.92 - 1.02) | (0.95 - 1.04) | (0.97 - 1.06) | (0.94 - 1.03) |
| COVID-19 Stringency Index (from 0 to 100, with 100 = strictest)) | 1.00 | 1.00* | 1.00* | 1.00*** | 1.01*** | 1.01*** |
|  | (1.00 - 1.00) | (0.99 - 1.00) | (1.00 - 1.01) | (1.00 - 1.01) | (1.01 - 1.01) | (1.00 - 1.01) |
|  |  |  |  |  |  |  |
| Observations | 19,453 | 10,687 | 19,372 | 30,649 | 29,581 | 41,064 |
| Number of Individuals | 4,063 | 2,272 | 4,142 | 6,422 | 6,270 | 9,029 |
| Pseudo R² | 0.007 | 0.013 | 0.007 | 0.006 | 0.012 | 0.010 |

Odds Ratios are reported; 95% CI intervals in parentheses; *** p<0.001, ** p<0.01, * p<0.05, + p<0.10; the count score ranges from 5 to 25, with higher values reflecting more problems in the EQ-5D-5L dimensions; Listwise deletion was used to handle missing values.

Supplementary Table 8. Drop-out analysis: Comparison of sociodemographic characteristics/EQ-VAS at baseline between individuals who participated in all nine waves (complete data) and individuals who only participated in wave 1

|  | Individuals who participated in all nine waves (n=626) | Patients who dropped out after wave 1 (n=2,341) | p-value |
| --- | --- | --- | --- |
| Age | 52.6 (13.6) | 39.4 (16.1) | <.001 |
| Gender |  |  | .05 |
| Male | 325 (51.9%) | 1114 (47.6%) |  |
| Female | 301 (48.1%) | 1227 (52.4%) |  |
| Educational level |  |  | <.01 |
| Low | 155 (25.0%) | 702 (30.7%) |  |
| Middle | 318 (51.2%) | 1000 (43.7%) |  |
| High | 148 (23.8%) | 584 (25.5%) |  |
| Professional Group |  |  | <.001 |
| Health-related sector | 41 (6.5%) | 256 (10.9%) |  |
| Education | 42 (6.7%) | 204 (8.7%) |  |
| Food retail | 22 (3.5%) | 179 (7.6%) |  |
| Research | 8 (1.3%) | 94 (4.0%) |  |
| Other | 513 (81.9%) | 1608 (68.7%) |  |
| Perceived income difficulties |  |  | <.001 |
| With great difficulty | 57 (9.1%) | 316 (13.5%) |  |
| With some difficulty | 222 (35.5%) | 907 (38.7%) |  |
| Fairly easily | 254 (40.6%) | 868 (37.1%) |  |
| Easily | 93 (14.9%) | 250 (10.7%) |  |
| EQ-VAS | 74.6 (20.9) | 76.2 (21.3) | .10 |

Mean (SD) or N (%) are shown, as appropriate. P-values are based on Chi²-tests or independent t-tests, as appropriate.
